# Report of the Madras Medical Board on Epidemic Cholera. Extracted Verbatim from the Original Document, Drawn up by Order of Government, 1824

**Published:** 1832-04-01

**Authors:** 


### XIII.

### REPORT OF THE MADRAS MEDICAL BOARD ON EPIDEMIC CHOLERA.†

(*Drawn up by Order of Government*, 1824.)

WHEN the Epidemic Cholera seemed to have completed its progress throughout the territories of this Presidency, it was intimated by Government to the Medical Board, that the interests of the medical science and of humanity, would probably be promoted by a history of that disease, as it had appeared amongst the population, as well as

† As the Report of the Madras Medical Board is the latest of the Indian Reports (1824), and consequently had the advantage of the Reports from Bombay and Bengal, so it is, by far, the most valuable. We could not do a greater service to the profession and to the public than by re-publishing this most important document. At the end of the article we shall give a tabular analysis of all the individual Reports from which the aggregate was compiled.

☞ We beg particularly to state, that this article is verbatim et literatim from the Madras Report (with the omission of some tabular returns and extraneous matters, for which we had not room), and not a *review* of the Report.—EDITOR.

amongst the troops; with an account of the various modes of treatment, which had been at different times adopted by different Medical Officers at their respective stations, and the success with which they had been attended.

The Honorable the Court of Directors have also subsequently ordered, that the particulars of the progress of Cholera through these territories, and the different modes which had been adopted in the treatment of persons afflicted with it, should be prepared and printed. In making this communication the Honorable Court stated, *that they had caused the reports which had been drawn up under the superintendence of the Bengal and Bombay Medical Boards to be circulated amongst the most eminent of the Faculty in England:* and the Court expressed their hope, that the circulation of these documents, together with that of a similar report from this Presidency, might tend to produce opinions and advice calculated to check the ravages of this afflicting malady. It is in pursuance of these instructions, that the present report has been prepared.

In addition to the measures for collecting professional information, which the ordinary discipline of the service implies, the Medical Board did not neglect, upon the first appearance of the epidemic cholera, specially to call the attention of all Medical Officers on this establishment, to the necessity of contributing such remarks respecting the disease as their opportunities for observation might enable them respectively to do; and it is a just tribute to the profession, which is gladly and gratefully awarded, to state, that the call has been fully answered by *almost every member of it.* A mass of information has thus been furnished, which leaves to the writer of this report little else than the labour of selection and arrangement. Many valuable communications have been received in a condition, fitting them to be at once offered to the public: and these will accordingly be found under the proper head in this report. Others, forming indeed a great proportion of the whole, though intrinsically valuable, and extremely interesting, are yet drawn up in a desultory manner, and in an insulated form, which precludes the propriety of their publication. In disposing of the contributions of the latter description, the utmost care has been taken to embody the facts and observations contained in them, under the proper heads of pathology and therapeutics. No observation has been set aside *merely* as being improbable; no theory absolutely rejected *merely* as being untenable: for our present state of knowledge with regard to cholera did not appear to warrant the absolute rejection of any medical opinion concerning it. Every alleged fact, and distinct theory, therefore, which have been recorded, find a place in these pages; and are thus left to be tried by the test of time and experience. So scrupulous indeed have the Medical Board been in the exercise of their authority, throughout the course of this destructive disease, that, even at the hazard of incurring the imputation of empiricism, they have given currency to the notice of remedies, and sanctioned their trial, although the exhibition of them might be little supported by any received system of medicine.

It would doubtless in some respects, have been more desirable in an official report, to have published every communication as it had been received, and with the name of its author, rather than to offer a digest of them: but, independently of the objections that have just been assigned, there existed another, which was, the great uniformity of these reports: for, though all Medical Officers have not been equally fortunate in their opportunities of observation, nor possessed of equal leisure, and an equal degree of zeal in the cause of science, yet by far the greater proportion of the reports, though occasionally relieved perhaps by a diversity of opinion on the *theory*, still present, in respect to the *history* of the disease and general plan of *treatment*, a repetition, which it would be altogether unprofitable to publish. It is perhaps more necessary to offer some apology to those Gentlemen whose reports, written in haste, and on the first appearance of the disease, are now published at this late period: and, had our knowledge of its nature, and method of cure kept pace with the extended means of observation, which the lapse of time has unhappily too amply afforded, there might have been good reasons for objection. The best apology, however, will be found in the reports themselves, which even when contrasted with those written at later periods, and under circumstances in every respect more favourable, will still be found to present most interesting matter, and to redound greatly to the credit of their authors, many of whom are now no more. Whenever supplementary remarks have been received from the same writer, they have been appended to his original communication.

Although no paper is inserted in this report, which is not official, or authenticated, nor any thing advanced, unless founded on materials exclusively of that nature, yet it is nevertheless to be remembered, that medical *facts*, are too often merely medical opinions; their authenticity, in reference to the views of the narrator, is undoubted: but liable, as even the most deliberate and sagacious observers are, to error and misconception, we are bound to receive with due caution and circumspection any *facts* or *opinions*, which are palpably at variance with general experience. It is presumed, however, that the report will be found upon the whole to convey a very considerable and unexceptionable mass of evidence respecting the nature of that species of cholera, which has so long afflicted the Indian community: and, while we sincerely hope that the actual presence of the disease in Britain will never afford more immediate means of judgment, we would still echo the sentiment of the Honourable Court, that the eminent of the Faculty there, may be more successful in their researches into its nature, cause, and cure, than their brethren in this country have been.

While the constitution of the Company's Medical Service is singularly well adapted for the collection of information, this advantage has almost been rendered a dead letter from the want of periodical printed reports; a want the more especially felt in a country where the change of functionaries is rapid and perpetual, and, consequently, where individual or personal knowledge is of less public avail. The evil here complained of has been strikingly exemplified in the instance of cholera, which, on its first appearance, was considered to be merely a severe, or unusual form of the disease known by the name of cholera morbus. This idea, however, being soon, and perhaps too hastily, abandoned, a persuasion was then almost universally entertained, that it was in fact *a new disease:* and, with a strange inconsistency, an expectation seems immediately to have arisen, that the nature of this new disease was more perfectly discoverable than the nature of those diseases, which have baffled the experience of ages; and farther, that a specific cure for it, was no less an object of easy attainment. Hence arose that diversity of opinion, and variety of practice in cholera, which at first generally prevailed, and which, it is feared still continues to exist to a very considerable degree. When it was found that a complaint, similar in all its symptoms, had been described in several European publications on diseases of India, the knowledge of the circumstance was too confined, and came too late to do away the evil, which had arisen. The Indian community, including the Medical profession, were taken, as it were, by surprise: the disease, if not new in reality, was new at least to them; and the pre-existence of cholera, in a form no wise different from that which it has on this occasion assumed, has been somewhat reluctantly admitted, and too much kept out of sight in reasoning on its pathology.

[*Appearance of Cholera in* 1787 *at Arcot.*] This state of things, however, cannot be imputed to the want of foresight in our predecessors; since we find the following notice entered in the proceedings of the Medical Board of this Presidency, under date the 29th November, 1787, but which, being confined to a manuscript entry in the records of an office accessible only to its members, it became, as has been observed, a dead letter.

"A disease having in October last prevailed at Arcot similar to an Endemic that raged amongst the natives about Paliconda in the Ambore valley in $\frac{1769}{1770}$ in an army of observation in January 1783, and in the Bengal detachment at Ganjam in 1781, and several other places at different times, as well as Epidemic over the whole coast in 1783, under the appearance of Dysentery, Cholera Morbus, or Mordyxim, but attended with spasms at the præcordia, and sudden prostration of strength as characteristic marks; seeing this Board is ordered to be a record, the Physician General recommends as a guide to future practitioners, that a letter from Mr. Thompson, Surgeon of the 4th Regiment, containing an account of the dissection of one of the patients who died of the disease, describing the state of the viscera, may be entered on the face of the proceedings, together with two letters from Mr. Duffin, Head Surgeon at Vellore; and one from Mr. Davis, Member of the Hospital Board, containing an account of the causes, symptoms, and successful treatment, of the sick, by the use of the hot-bath, and fomentations, supporting the vis vitæ with wine, &c., and removing the putrid colluvies from the intestines. The Hospital Board, sensible of the advantages that may result to the service from the mode proposed by the Physician General, direct their Secretary to enter the Letters he has mentioned as follows."*

[*Noticed by Bontius in* 1629.] The Dutch Physician, Bontius, who wrote in the year

* Vide page 506, where this document is embodied in the Report.—Ed.

1629, at Batavia, thus describes cholera morbus. "Besides the diseases above treated of as endemic in this country, the cholera morbus is extremely frequent; in the cholera, hot, bilious matter, irritating the stomach and intestines, is incessantly, and copiously discharged by the mouth and anus. It is a disorder of the most acute kind, and therefore requires immediate application. The principal cause of it, next to a hot and moist disposition of the air, is an intemperate indulgence of eating fruits; which, as they are generally green, and obnoxious to putrefaction, irritate and oppress the stomach by their superfluous humidity, and produce an æruginous bile. The cholera might, with some degree of reason, be reckoned a salutary excretion; since such humours are discharged in it, as, if retained, would prove prejudicial. However, as by such excessive purgations the *animal spirits are exhausted, and the heart, the fountain of heat and life, is overwhelmed with putrid effluvia, those who are seized with this disorder generally die, and that so quickly, as in the space of four and twenty hours, at most.*

Such, among others, was the fate of Cornelius Van Royen, steward of the Hospital of the sick, who being in perfect health, at six in the evening, was suddenly seized with the cholera, and expired in terrible agony and convulsions before twelve o'clock at night; the violence and rapidity of the disorder surmounting the force of every remedy. But if the patient should survive the period above-mentioned, there is great hope of performing a cure.

*This disease is attended with a weak pulse, difficult respiration, and coldness of the extreme parts; to which are joined, great internal heat, insatiable thirst, perpetual watching, and restless and incessant tossing of the body. If together with these symptoms, a cold and fetid sweat should break forth, it is certain that death is at hand.*"

In treating of the "Spasm," this author gives the following account. "The disorder of the Spasm, almost unknown with us in Holland, is so common in the Indies, that it may be reckoned among the popular and endemic diseases of the country. The attack of it is sometimes so sudden, that people become in an instant as rigid as statues; while the muscles either of the anterior or posterior part of the body, are involuntarily, and violently contracted. A terrible disorder! which, without any primary defect of the vital or natural functions, quickly precipitates the wretched sufferer in excruciating torment to the grave; totally deprived of the capacity of swallowing either food or drink. *There are, likewise, other partial Spasms of the limbs*; but these being more gentle and temporary, I shall not treat of them.

People affected with this disease look horribly into the face of the by-standers (truculente admodum astantes intuentur) especially, as often happens, when the cynic spasm comes on, and both the cheeks are drawn in convulsion towards the ears; a red and green colour is reflected from the eyes and face; (ruber et viridis color ex oculis et facie *oritur*), the teeth gnash; and instead of the human voice, a rude sound issues forth of the throat, as if heard from a subterraneous vault; so that to those unacquainted with the disorder, the person appears to be dæmoniac."

In speaking of cholera, Bontius no where mentions the *colour* of the matters evacuated. He talks indeed of æruginous bile: but that would appear, from the context, to refer to its assumed acrimonious quality, rather than to any sensible property; and we shall presently see that practitioners of much later times dwell greatly on the supposed bilious and irritative nature of the evacuations, when it is pretty evident, that they were merely speaking hypothetically. His descriptions indeed are not at all full: for, though he does not mention spasm as a symptom of cholera morbus, he states that Cornelius Van Royen expired in *convulsions*, within six hours from an attack of it. Still, in his description of cholera, where "the heart is overwhelmed," where "those who are seized with the disease generally die," and that within twenty-four hours at most; and in his enumeration of symptoms as marked in italics, every one, familiar with the epidemic cholera as it has prevailed in this country, will probably admit, that he has truly pourtrayed that disease, and no other.

Although Bontius has treated of "the Spasm," and of "the Cholera Morbus," under separate chapters, it is highly probable that these disorders were one, and the same.

It would seem that he has considered the tonic spasm as idiopathic, and the clonic spasm as symptomatic, yet it is evident by the expression, "there are likewise other partial spasms of the limbs," that both these forms of spasm existed in the same patient, a fact which is amply confirmed by innumerable observations in the present epidemic. If it be objected that he does not mention the usual symptoms of cholera as occurring in "the spasm," it may be answered, that neither does he mention the state of the skin, of the pulse, nor of respiration, which functions it is impossible to suppose remained unaffected in such a commotion of the system.

The edition of Bontius, which has been quoted from, is an English translation published in London, 1769; but, from the passages in the original, as inserted in parenthesis, it is evident that the translation is not quite correct. The expressions, especially, of the eyes and face "*reflecting*" a red and green colour can only be intelligible by supposing, that the former were suffused with blood, and the latter changed to that ghastly and cadaverous hue, so familiar to us all in the collapse stage of cholera.

[*By Dr. Paisley in* 1774.] The next notice, in point of time, which we find of cholera, is in the copy of a Letter written by Dr. Paisley at Madras, dated 12th February, 1774, as given by Curtis, in his publication on the diseases of India. Dr. Paisley says, "I am favoured with yours, and am very happy to hear you have caused the army to change its ground; for there can be no doubt, from the circumstances you have mentioned, that their situation contributes to the frequency and violence of the attacks of this dangerous disease, which is as you have observed, a true cholera morbus, the same they had at Trincomale. It is often epidemic among the Blacks, whom it destroys quickly, as their relaxed habits cannot support the effects of sudden evacuations, nor the more powerful operation of diseased bile.

The first campaign made in this country the same disease was horridly fatal to the Blacks; and fifty Europeans of the line were seized with it. I have met with many single cases since, and many of them fatal or dangerous, of different kinds arising from putrid bile being disturbed by accidental causes, or by emetics or purgatives exhibited before it had been blunted or corrected."

Dr. Paisley does not give any particular description of the disease: and though he dwells much on the putridity, and acrimony of the bile, he does not allude to the colour or appearance of the evacuations. He observes that "when it (the cholera) is epidemic here, it is totally a disease of highly putrid bile, which operates on the system as poison, and brings on *sudden prostration of strength, and spasms over the whole surface of the body*." "In relaxed habits, when the *pulse sinks suddenly*, and brings on immediate danger, the same method must be pursued but with more caution." The letter is quoted by Curtis as referring to the cholera morbus, or Mort de Chien: and these extracts will probably be deemed sufficient evidence of the correctness of the reference.

It is highly important to remark, that Dr. Paisley here speaks of the disease as being "*often Epidemic*;" that it prevailed in that form in the "first campaign," and affected both the Europeans and Natives. The particular periods here alluded to are not known, but we have seen, by the extract from the records of the Medical Board, that cholera raged as an epidemic, in 1769, or 70.

[*By Sonnerat from* 1774 *to* 1781.] Sonnerat, whose travels in India embrace the period between 1774 and 1781, speaks of a disease on the Coromandel Coast, in all respects resembling cholera, and he notices it as "an *epidemical disorder which reigns*." His account of it is this.

"There is also another epidemical disorder, which reigns, and in twenty-four hours, or sometimes less, carries off those who are attacked. It never appears but in cold weather."

"Debauchees, and those who have indigestions, are attacked with a looseness, or rather with an involuntary flux of the excrementary matter become liquid, but without any mixture of blood. They have no remedy for this current of the bowels,* which they call a sharp flux, but leave the cure to the care of nature."

"The flux of this kind which reigned some years ago, spread itself in all parts, making great ravages: above sixty thousand people, from Cherigam to Pondicherry, perished. Many causes produced it. Some were attacked for having passed the night and slept in the open air; others for having eat cold rice with curds; but the greater part for having eat after they had bathed and washed in cold water, which caused an indigestion, an universal spasm of the nervous kind, followed by violent pains and death, if the patient was not speedily relieved. This Epidemical disorder happened during the Northerly

* Probably "cours de ventre," in the original. The edition here quoted is a translation by Francis Nagnus, Calcutta, printed 1733.

winds in December, January, and February; when they ceased the malady disappeared. The symptoms of this disorder were a watery flux, accompanied with vomiting and extreme faintness, a burning thirst, an oppression of the breast, and *a suppression of urine.* Sometimes the deceased felt violent cholicky pains; often lost his speech and recollection, or became deaf, *the pulse was small and concentered,* and the only specific which Choisel, a foreign Missionary, found, was treacle and Drogue amere. The Indian Physicians could not save a single person."

"There is great reason to imagine that the perspiration being stopped and reflowing into the mass of blood, by finding its way to the stomach and bowels, occasioned the vomiting, which terminated by this flux."

"That which followed, two years after, was the most dreadful. It did not proceed from the same cause as the first, as it began in July and August: it first shewed itself by a watery flux, which came in an instant, and sometimes cut the deceased off, in less than four and twenty hours. Those who were attacked had thirty evacuations in five or six hours; which reduced them to such a state of weakness that they could neither speak or move. *They were often without pulse; the hands and ears were cold; the face lengthened; the sinking of the cavity of the socket of the eye was the sign of death*; they felt neither pains in the stomach, cholics, nor gripings. The greatest pain was *a burning thirst.* Some brought forth worms by stool; others by vomiting. This cruel pestilence affected all the Casts in general, but particularly those who eat meat, as the Parias. The Native Physicians succeeded no better in their treatment of this disorder, which was again renewed during the North-winds."

It is by no means easy to determine the precise dates of the epidemic visitations of cholera alluded to in these extracts, as prevailing, in the first instance, "*some years ago,*" and in the second, "*two years after.*" It is, however, reasonable to suppose, that a disease, which "spread itself in all parts," and carried off "above 60,000 people from Cherigam to Pondicherry," would not have been passed over without some special notice by Dr. Paisley, in his letter dated 1774 already quoted, had it occurred prior to that date. The presumption seems to be, that Mons. Sonnerat described invasions of epidemic cholera which took place subsequently to the year 1774. That they were certainly considerably prior to that epidemic, which is stated in the records of the Medical Board to have prevailed "over the whole Coast in 1783," is evident, from the date of the work; and consequently, *when viewed in reference to other authorities, it is obvious, that cholera maintained its influence, with little apparent interruption, from a very remote period, down to a date comparatively modern.* Sonnerat notices the "term mort de chien" as being used in India, but applies it to "indigestions," which "are very frequent," and from which "many have died suddenly."

[*Cholera observed at Mauritius in* 1775, *and in* 1819.] It appears from the report of a Committee of British Medical Officers at the Mauritius, which was assembled in the month of November, 1819, under the authority of the Government, in order to examine into the nature of the epidemic disease which then prevailed at that Island, that the epidemic cholera was not unknown there. The following is an extract from the report. "The Committee request to say, that they have not, either in this Island or elsewhere, met with a disease possessing the characters of that which now prevails; but that, from the reports of several individuals, some of whom belong to the medical profession, it does appear, that a disease, most strongly resembling in its symptoms, progress, and termination, that now under consideration, did for some time prevail in this colony in the year 1775."

The symptoms which are detailed by the Committee, as characterising the epidemic of 1819, sufficiently indicate the identity of that disease, with the form of cholera, which prevailed at the same period, and still continues, on the continent of India. "The symptoms, in the two cases alluded to, perfectly corresponded with those of the numerous instances of the disease, which have since occurred. Those more particularly characteristic of the disease are sudden and excessive prostration of strength, with sinking of the pulse; extreme coldness of the surface of the body, which is covered with cold viscid perspiration; and a distressing uneasy sensation in the abdomen, the progress of which has generally carried off the patient in the space of a few hours."

Dr. Burke, the chief medical officer in the Island makes the following observation in his letter transmitting the report of the committee. "*A similar disease prevailed in this island in* 1775, *after a long dry season, &c. the symptoms, fatal and sudden effects,*

*and duration, of the disease would seem to be exactly the same.* A hurricane put a stop to its ravages, which continued for probably two months, and caused a great mortality particularly among the Blacks and people of colour."

But it is necessary to state, that a committee of French Medical Gentlemen, who were assembled under similar circumstances with the British Committee, make no mention of the epidemic visitation of 1775. Assuming, however, the circumstance to be true, it is highly worthy of remark, that while, as we have shewn in the preceding pages, the Indian continent suffered under cholera, about that period, viz. 1775, the disease had then also extended to that remote Island.

[*At Ganjam, in* 1781.] Cholera appears to have manifested itself pretty extensively as an epidemic in 1781; its appearance on this occasion is thus noticed in the report on cholera by Mr. Jameson, Secretary to the Calcutta Medical Board. "A Division of Bengal Troops, consisting of about 5,000 men, was proceeding, under the command of Colonel Pearse of the Artillery, in the Spring of 1781, to join Sir Eyre Coote's Army on the Coast. It would appear, that a disease resembling cholera had been prevalent in that part of the country, (the Northern Circars), some time before their arrival; and that they got it at Ganjam on the 22d March. It assailed them with almost inconceivable fury. Men previously in perfect health dropt down by dozens; and those even less severely affected were generally dead or past recovery within less than an hour. The spasms of the extremities and trunk were dreadful; and distressing vomiting and purging were present in all. Besides those who died, above five hundred were admitted into hospital on that day. On the two following days, the disease continued unabated, and more than one half of the Army was now ill." In a note it is added, 'The occurrence of the disease on this occasion is noticed in a letter dated 27th April, 1781, from the Supreme Government to the Court of Directors; and the destruction which it caused in this detachment mentioned in terms of becoming regret.'

After adverting to its progress in the Circars, the letter thus proceeds: 'The disease to which we allude, has not been confined to the country near Ganjam. It afterwards found its way to this place (Calcutta); and after chiefly affecting the native inhabitants, so as to occasion a great mortality during the period of a fortnight, it is now generally abated, and pursuing its course to the northward.' It would have been interesting to have traced this disease, as it seemed to have put on the epidemical form, but every attempt to discover its further progress has proved fruitless.

[*Noticed by Curtis, in* 1782.] From this period, up to the year 1787, and perhaps even to 1790, the cholera would appear to have existed epidemically, in various parts of India. Curtis states, that the fleet, in which he served, joined Sir Edward Hughes's squadron at Madras, in the beginning of 1782; in May of that year, his ship, the Seahorse, arrived at Trincomalee, and he says, "The mort de chien, or cramp, I was also informed by the attending Surgeon, had been very frequent and fatal among the seamen, both at the hospital, and in some of the ships, particularly in the Hero and Superb." The Seahorse had no case of the disease till the 21st of June, when between that day and the 25th they had eight cases.

"In every one of the eight cases the symptoms were so much alike, both in order and degree, that a description of any one would answer almost equally well for every other. Any difference that took place was in the suddenness of the attack, or the rapidity with which the symptoms succeeded each other. In all of them the disease began with a watery purging, attended with some tenesmus, but with little or no griping. This always came on some time in the night, or early towards morning, and continued some hours, before any spasms were felt; and slight affections of this kind being very common in the country, the patients seldom mentioned them till they began to be more severe, and extended to the legs or thighs. *This purging soon brought on great weakness, coldness of the extremities, and a remarkable paleness, sinking and lividity of the whole countenance.* Some at this period had some nausea, and retching to vomit, but brought up *nothing bilious.* In a short time the spasms began to affect the muscles of the thighs, abdomen, and thorax, and lastly they passed to those of the arms, hands and fingers; but I never saw, then or afterwards, those of the neck, face, or back at all affected. The rapidity with which these spasms succeeded the first attack, and their severity, especially as affecting the muscles of the thorax and abdomen, denoted in general the degree of danger in the case. The affection is not, as in *tetanus,* confined to a single muscle, or to a certain class of muscles only. Neither does it, as in the spasmus clonicus, move and agitate the members. It is a fixed cramp in the belly of the muscles, which is gathered up into a hard knot, with excruciating pain. In a minute or two this relaxes, is again renewed, or the affection passes to others, leaving the miserable sufferer hardly an interval of ease; and, lastly, it passes from one set to another; from those of the inferior extremity to those on the upper parts, leaving the former free. The patients complain much of the pain of these cramps; think they obtain some relief from friction of the parts, and cry to their companions to rub them hard. As the disease proceeded, the countenance became more and more pale, wan, and dejected; *the eyes became sunk, hollow, and surrounded with a livid circle. The pulse became more feeble, and sometimes sank so much as not to be felt at the wrist, in two or three hours after the spasms came on.* But so long as it could be felt, it was but little altered in frequency. If the spasms happened to intermit, it would sometimes rise a little, and the countenance assume a better look. The tongue was generally white, and more or less furred towards the root; the patients had all *great thirst, or rather a strong desire for cold drinks; but there was no headache or affection of the sensorium commune throughout.*"

"The coldness of the extremities, which was perceptible from the very first, continued to increase, and spread over the whole body, but with no *moisture* in the skin till the severity of the pain and spasms forced out a clammy sweat, which soon became profuse. *The hands now began to put on a striking and peculiar appearance. The nails of the fingers became livid, and bent inwards; the skin of the palms became white, bleached, and wrinkled up into folds, as if long soaked in cold water;* the effect, no doubt, of the profuse cold sweat, which is one of the most pernicious and fatal symptoms of the disease, both from the effect it has in such a climate, of exhausting the strength, and in abstracting heat from the system. In some of the present cases, and in many others after this, we had recoveries from the severest degrees of spasmodic affection; even where the pulse had been for hours completely lost at the wrist, and the body perfectly cold; but never of any who had these profuse cold clammy sweats, and where the hands had put on this appearance."

"All this while the purging continued frequent, and exhibited nothing but a *thin watery matter or mucus.* In many, the stomach became at last so irritable, that nothing could be got to rest upon it; but every thing that was drank was spouted out immediately; without straining or retching. *The countenance and extremities became livid, the pulsations of the heart more quick, frequent and feeble;* the breathing began to become laborious and panting; and, in fine, the whole powers of life fell under such a great and speedy collapse, as to be soon beyond the power of recovery. In this progression, the patient remained from three to five or six hours from the accession of the spasms; seldom longer. These began at last to abate, but with more internal oppression, great jactitation, panting and gasping for breath, from the diminished action of the respiratory organs; for there were no marks of oppression or effusion on the lungs; and the motion of the heart, so long as it could be felt, became more and more quick and irregular, till death came at last to the relief of the miserable sufferer." Sometime before that event took place, *the spasms gradually abating, left the sufferers entirely, and so much possession of their faculties did they retain, that they would continue to talk sensibly to their messmates, to the last moment of their life, even when the whole body had become perfectly cold, and all pulsation of the heart had ceased for a long time to be distinguishable.*"

"About the middle of July, 1782, I entered on duty at Madras Hospital. Here, again, I had occasion to see many more cases of the mort de chien. It was frequent in the fleet in the month of August, and beginning of September, the season at which the land wind prevails on this part of the coast. We had some cases in the hospital in the end of October, and in November after the monsoon, but few in comparison."

[*Also by Girdleston.*] Although cholera would thus appear to have been of limited prevalence in the Naval Hospital at Madras, in October, 1782, its influence was most severely felt at that period by the newly-arrived troops from England, as stated by Girdleston in his Essay on Spasmodic affections of India.—He observes, "spasms were the first disease which appeared amongst the troops who arrived at Madras in October, 1782, under the command of Major General Sir John Burgoyne. More than fifty of these fresh men were killed by them within the first three days after they were landed in that country, and in less than a month from that time, upwards of a thousand had suffered from attacks of this complaint."

"The symptoms which commonly first presented themselves were coldness of the surface of the body, especially of the hands, feebleness of the pulse, and spasmodic contractions of the lower extremities, soon extending to the muscles of the abdomen, diaphragm, and ribs. As the spasms advanced, the muscles might be seen to assume the rigidity of cartilages; sometimes causing the body to remain immoveably extended, sometimes bending the trunk through its whole length, anteriorly; and sometimes though seldomer, backwards. The parts in which the spasms began generally remained rigid; but those which were subsequently seized with them, had momentary intermissions of the contractions; the only intervals of relief experienced by the patient from the most tormenting pains. *The hands and feet then generally became sodden, with cold sweat, the nails livid, the pulse more feeble and frequent, and the breath so condensed as to be both seen and felt, issuing in a cold stream at a considerable distance. The thirst was insatiable, the tongue whitish, but never dry*; vomitings became almost incessant; the spasms, cold sweats, and thirst, increased with the vomitings; which last, if not checked, soon terminated the existence of the patient." "In this manner, most commonly was the succession of phœnomena; but often they were so rapid in their attack, that they seemed to seize the patient all in conjunction instantaneously."

"In some few, the extremities remained warm; in others also the spasms were only clonic or convulsive. Some died in the first hour of the attack; others lived a day or two with remissions; when they died, either of universal spasms or an apoplexy. *On dissection of the bodies after death, it appeared that no injury had been sustained by the brain, liver, gall-bladder, stomach or heart.* The prognosis of this disease is formed with greater certainty from the warmth or coldness of the extremities, than from either the universality of the spasms, or the frequency or steadiness of the pulse. Thus if the spasms were ever so general, with warmth of the extremities, there was no immediate danger: on the contrary if the spasms were ever so trifling, with coldness, there was every danger to be feared."

Girdleston, like Bontius, treats of the "Spasms" as an idiopathic disease; yet it is obvious from his observations on the prognosis, that spasm was merely a symptom, and one of secondary importance. He has not noticed purging, and from the casual way in which vomiting is mentioned, it seems doubtful whether we are to consider purging to have been inadvertently omitted, or that it was really not present, as has often been observed to be the case on late occasions. It is accordingly assumed that the "spasm," described by Girdleston, was in fact the spasmodic cholera, or mort de chien of Curtis.

It is also noticed in the Bengal Report, that in the month of April, 1783, cholera destroyed above 20,000 people, assembled on occasion of a festival at Hurdwar; but it is said not to have extended to the neighbouring country. All these authorities would seem accordingly to establish the fact of the prevalence of cholera in India; and especially of its existence during the period extending from 1769-70 to 1787, when we find the first notice of the disease in the records of this Office as given in the extracts, page 239, and which we now come to consider.

[*Dr. Duffin's Account of it, at Vellore, in* 1787.] Dr. Duffin, in a letter dated the 28th October, 1787, says, "I returned yesterday from Arcot, where I had an opportunity of seeing the situation of the sick. The cholera morbus rages with great violence, with every symptom of putrescency, and so rapid in its progress, that many of the men are carried off in twelve hours' illness." Dr. Duffin considered the disease to depend on putrid bile; he recommended castor oil, external heat, frictions, and the internal exhibition of warm cordial drinks, as the plan of treatment he had always found successful. In a subsequent letter, dated the 3d November, he enters more fully on the nature of the disease. "The symptoms were generally pretty much the same in all I have seen, only the violence of the spasms was greater according to the stamina of the patient, and the quantity of putrid matter in the primæ viæ. They generally are seized with a nausea, frequent heats, and chills, a dryness of the skin, and numbness and uncommon sensation, as they express it, in different parts of their body. Then came on cold sweats, severe gripings, and mostly a purging of bilious colluvies, appearing often in a ferment like yeast, and not unlike it in colour, with a putrid offensive smell, retchings to vomit, often bilious, and at other times scarce any thing is brought up, but the liquor that is drank; an intense thirst, oppression on the præcordia, with difficulty of breathing, frequently the spasms begin with the first attack, though sometimes they only appear as the disease advances, and then generally affect the lower extremities, afterwards the abdominal muscles; and the whole system becomes convulsed. The pulse from the first sinks, and at times is scarce to be felt; profuse clammy cold sweats, and a pallid hue overspread the body; the countenance ghastly, the eyes sunk, and the voice scarcely to be heard, with great dejection. The tongue in general moist, till near the close of the disease, when it becomes dry and foul, and the breath offensive; the urine generally pale, and in small quantity."

It is to be observed, that Dr. Duffin, at the period in question, was stationed at Vellore, about 14 miles from Arcot; and that his description of the cholera could not be founded on any lengthened observation of the cases at the latter station, since he only made a very short visit to it. There seems some reason to doubt, therefore, whether he was not describing partly what he saw at Arcot, and partly what he had more experience of at Vellore, where the cholera was then also raging, but not in a very dangerous degree. His confident allusion to the bilious nature of the contents of the primæ viæ, and the success of castor oil in curing the disease, may lead us to suppose that at Vellore, he had in fact to contend chiefly with the cholera morbus as it is commonly termed, not with the epidemic or spasmodic cholera. This conclusion is supported by a reference to the sick returns, which happen in this instance to be somewhat less meagre and imperfect than they generally are found to be at that distant period.

It appears that, during the month of October, 1787, twenty-two Europeans were admitted into hospital with "cholera morbus" at Vellore, and two Natives; of whom it cannot be ascertained that any died, for the returns of that period do not shew the disease from which the casualties arose; however, only two Europeans died during that month at Vellore from any disease, and not one Native. At Arcot, on the contrary, 35 Europeans are entered in the sick returns, under the head of "cholera morbus," in October, 1787, but no Natives; and 25 Europeans died that month, a number which falls short of what Dr. Davis distinctly attributes to cholera alone. In November, 45 Europeans are returned at Vellore, under the head of "cholera morbus," and 1 Native; only one European died that month at Vellore. At Arcot, 17 Europeans are returned in November as ill with "cholera morbus;" only one death took place in all, but during this month it seems to have slightly affected the Natives, 12 being returned, of whom none died; there were at this time four Regiments of Native Cavalry quartered there. It seems reasonable, therefore, to infer, that the disease prevalent at Arcot, and described by Mr. Davis as "a spasmodic affection of the nervous system," was not the same, in general, with that which existed at Vellore, unless we impute a degree of efficacy to castor oil which can hardly be admitted.

[*Dr. Davis's Account of it at Arcot in* 1787.] Mr. Davis, a member of the then Hospital Board, appears to have been deputed from Madras, to investigate the nature of the sickness which prevailed at Arcot. In his report to the Board, which is dated the 29th November, 1787, he states as follows. "I found in what was called the epidemic hospital, three different diseases, viz. patients labouring under the cholera morbus, an inflammatory fever, with universal cramps; and a spasmodic affection of the nervous system distinct from the cholera morbus. I understood from the regimental Surgeon, *that the last disease had proved fatal to all who had been attacked with it;* and that he had already lost seven and twenty men of the regiment in a few days. Five patients were then shown me, with scarce any circulation whatever to be discovered; their eyes much sunk within their orbits; their jaws apparently set, their bodies universally cold, except at the præcordia, and their extremities livid. Mr. Pringle observed that these five men were attacked on the 26th October, that Mr. Duffin had seen them, and had recommended castor oil to be administered, &c. &c."

He then goes on to say, "finding on the day of their attack, the rectum had discharged its contents in the action of straining to vomit without being able to bring any thing up, I directed a stimulant injection to each of these patients, which produced a copious discharge of fæces, without any bilious induration (indication?) whatever." Having prescribed some antispasmodic medicines, he says, "from all which, I had the pleasure to observe, that in four and twenty hours after my first visit, the spasms had totally subsided, the patient's voice, which all along had been so low as scarce to be heard, was returned almost to its natural state; the pulse that was imperceptible, full and even." After ordering some carminative purgatives he observes, "I attended to the operation of these respective medicines and could discover no bilious indication in the whole system."

Two of the five patients having died in a few minutes after being taken out of a hot medicated bath, "upon dissection the duodenum was found distended with putrid air; the other intestines empty except the colon, and rectum, in which latter there were indurated fæces; the whole viscera sound, the gall-bladder turgid, but not diseased." !

Mr. Davis does not state the symptoms of the "inflammatory fever with violent cramps," farther than that the patient complained of a "tightness of the abdomen with a costive habit." The "cholera morbus" was distinguished by "spasms of the præcordia, and cramps of the extremities with bilious lientery, and a copious discharge from the stomach of a green, yellow and dark, coloured bile." During his residence at Arcot, upwards of sixty patients labouring under these three forms of disease were admitted, and only two or three deaths ensued. The dissection of a case is given, where, it is stated, "*the bladder was most singularly contracted, and did not exceed in size a large nutmeg*, yet without inflammation, or any apparent disease, except its contracted state."

[*Mr. Thompson's account of it, at Arcot and Trincomallee.*] Mr. Thompson, Surgeon, who was also sent to Arcot at the same time with Mr. Davis, observes, "This disease is exactly the same as prevailed at Trincomallee in the months of April and May 1782, when the season was very hot, and chill, the winds blowing from the land, and reaching some leagues to sea. The weather here at present is the same as I experienced at Trincomallee." Mr. Thompson also gives an account of a dissection where "the gall-bladder was exceedingly distended with bile, so much so as to appear protruded some inches below the liver, and to contain near six ounces of bile. No marks of putrescence in any of the abdominal viscera. *The urinary bladder quite empty and contracted to the size of a walnut*; the stomach and duodenum both empty of bile, and no appearance of inflammation in any part of the intestinal canal or peritoneum."

To persons familiar with the progress of cholera during late years, there can be little difficulty in understanding and reconciling the apparent discordances in the accounts just quoted. Many instances of the common cholera would seem to have occurred at Arcot, as well as at Vellore, where, it has been conjectured, this form of the disease chiefly prevailed. Some cases seem to have commenced with a degree of febrile excitement, an occurrence which has been occasionally observed in the present epidemic; or perhaps, these cases might be properly referred to a species of febrile affection with cramps, of which we have a very distinct history by Mr. Anderson, who observed the disease at Ellore in 1794, and styled it a "Causus;" lastly, that which Mr. Davis characterises as a "spasmodic affection of the nervous system distinct from cholera morbus," was no doubt the same low and dangerous form of the disease with which we have become too well acquainted in recent times.

The disease would seem to have lost its force at the period when Mr. Davis arrived at Arcot; for we find that the five cases, of the low form, which he first saw, had lingered from the 26th to the 29th; and few of the subsequent seizures proved fatal, which is quite analogous with our present experience.—Whether the bowels were less generally affected in that epidemic, or whether the means employed, and the prolongation of life for three days had given rise, in the cases in question, to fæcal formations, and to their accumulation in the large intestines, it is not easy, from the scantiness of our information, to decide. But, if any doubt could be entertained of the cases described being cholera, such as we have lately witnessed, the testimony of Mr. Thompson to their identity is conclusive, if we admit that the Mort de Chien of Curtis, which he states to have prevailed at Trincomallee at the time mentioned by Mr. Thompson, was really cholera.

[*Cholera noticed in* 1790 *in the Northern Circars.*] It is stated in the Calcutta report, that "Cholera was again very prevalent and destructive in a Detachment of Bengal troops marching through the Northern Circars, in the months of March, April, May and June of 1790. "The disorder was characterised by precisely the same symptoms which marked the late epidemic. It began with violent pain and spasm in the stomach and bowels; which were followed by purging, vomiting, and all the signs of extreme debility."

[*Noticed by Dr. James Johnson.*] The next account we have of cholera is to be found in Dr. Johnson's work on the diseases of Tropical Climates. It does not appear in that work, that cholera was then epidemical, but it would seem to have occurred pretty frequently, both on shore, and on ship board, chiefly in the vicinity of Trincomallee. The precise date is not mentioned; it is concluded, however, to have been about 1804. Dr. Johnson does not detail the symptoms with much minuteness, contenting himself with those occurring in one or two cases, and referring generally to Curtis's description of the disease, a pretty satisfactory proof that they were the same. A seaman on awaking after a debauch, repaired to the deck, and there again fell asleep, during the chilly part of the night. "About 4 o'clock in the morning, he awoke with a shiver and left the deck, but was soon seized with frequent purging and griping, his stools consisting of mucus and slime. Nausea and retching succeeded; nothing being ejected but phlegm, and the contents of the stomach. His pulse was now small, quick, and contracted; his skin dry, but not hot. About eight o'clock in the morning he began to feel spasms in different parts of his body, which soon attacked the abdominal muscles, and threw him into great pain. During these paroxysms, a cold clammy sweat would be occasionally forced out, especially in the face and breast. The extremities now became cold; his features shrunk; the stomach rejecting every thing, which was offered, either as medicine or drink. The abdomen and epigastrium all this time were distended and tense, with incessant watery purging and painful tenesmus. By ten o'clock his pulse could scarcely be felt: his breathing was oppressed and laborious, his eyes sunk, and the whole countenance singularly expressive of internal agony and distress. The extremities were cold, shrivelled, and covered with clammy sweats. The violence of the spasms now began to relax: and by eleven o'clock, or seven hours from the attack, death released him from his sufferings." "This may serve as a specimen of the worst form of that dreadful disease, which has obtained the appellation of "mort de chien," or the "death of a dog."

[*Cholera supposed to have been met with at various times since* 1787.] Since cholera has become familiar to the older practitioners here, many, perhaps all of them, recollect having met with insulated cases of that disease, as well as of sudden, and often fatal illness, which they, at the moment, could not well understand, and which consequently, proved extremely embarrassing. Such cases would no doubt be attributed by different practitioners to different causes, and be referred to different heads of disease according to the various states in which the patients were seen; and, perhaps some of them were considered to be merely anomalous instances of common cholera; but late experience has now very generally led to the opinion, *that they were in fact cases of spasmodic cholera.* The records of the Medical Board throw no light on this subject. The number of cases of the description alluded to, which may have entered the military hospitals, could not however, in all probability, have been great, without attracting observation. It might perhaps be thought that the necessity of classing the cases in the official returns would have led to their being detected by a bare inspection of these documents; but in the absence of any nosological arrangement, which then distinguished the returns, no difficulty would be experienced in disposing of them.

Sporadic cases of spasmodic cholera might naturally produce the impression that some poisonous matter had been swallowed, which other circumstances would contribute to render sufficiently plausible; for, it is notorious, that intoxicating liquors are prepared by the natives, and clandestinely sold to the European soldiery, which contain the most deleterious matters, and which often produce fatal consequences to those who drink them. The symptoms attending such cases are frequently very anomalous and perplexing. Although the natives are less prone to debauch in spirituous liquors, they are yet not altogether to be exempted from the reproach; and the notion of a poison having been swallowed, would in their case be rendered still more probable, from such occurrences being not unknown amongst them, and from our ignorance of the nature of the poisons which are used.

It must be admitted however, that very few cases either of sudden death, or poisoning, or cholera, are to be found in the returns; but it will be presently shewn that no positive conclusion can thence be drawn against the existence of spasmodic cholera prior to the year 1818, when it appeared epidemically in these territories; and that some at least of the cases designated as cholera in former times, were clearly of the spasmodic species.

[*Described by Mr. J. Wyllie in* 1814.] Mr. John Wyllie, in his report dated 20th July, 1818, (page 68) makes the following remarks. "Before concluding, I think it proper to add, that although I have never, before the late occasion, seen this peculiar disease prevailing as an epidemic, yet I have at various times met with single cases of it in the most aggravated form, and I am much mistaken, if I have not recorded two particular instances of it in my journal of the 1st Battalion 24th Regiment for the month of June 1814, under the names of Paramuttee, and Madaramooto, Sepoys." On referring to these cases, which have been preserved, Mr. Wyllie's conjecture seems to be fully confirmed: The first case is thus described. "Jaulnah, 19th June, 1814, P.M. ½ past 2. He is in a state of the most extreme exhaustion, unable to move or speak, features contracted; eyes sunk, half open, and of a dull lustre; countenance bedewed with a cold sweat; pulse low, skin cold. Has been vomiting and purging very frequently since 7, A.M. and had all yesterday been affected by a watery diarrhœa;" at 3, P.M. he "is greatly distressed by excruciating crampy pains of the thighs and legs;" at 9, P.M. "complains of thirst, tongue moist;" on the 20th he "continues very low; countenance still of a ghastly appearance;" "alvine discharge copious, alone of ash-coloured slime." The patient recovered. The next case is on the 24th June, 7, A.M. "Is in great distress from violent crampy pains of the muscles of the upper and lower extremities, but more particularly of the fingers; there is great prostration of strength; countenance ghastly; surface cold; pulse gone, much thirst. He had a very copious watery purging on him since 1 o'clock this morning. He attributes his complaints to having slept last night on the damp ground, and in the open air while on guard; at 9, A.M. "pulse just perceptible;" at 2, P.M. "slight giddiness, eyes red, says he has much appetite;" at 6, P.M. "one copious pale watery evacuation." This man also recovered, and both were treated with opium, and diffusible stimuli.

[*Also by Mr. Cruickshanks in* 1814.] Another incidental notice of cholera, has led to the discovery of that disease having prevailed to a remarkable extent, at the same time, and much in the same neighbourhood, as in the preceding instance. The late Mr. J. J. Duncan, in a report dated 1st September, 1819, after making some observations on the comparative advantages of dry and moist heat, externally applied, goes on to say, "In the month of June 1814, when the cholera appeared with great severity in the 1st Bat. 9th Regt. N. I. on its march from Jaulnah to Trichinopoly, I employed exactly the same plan of exciting heat, (heated sand), and found the greatest benefit resulting from it." "The disease in the 9th Regiment in 1814 resembled in every particular, (with the exception of the heat at the præcordia), the cholera at present so common, although it could not be called epidemic. The best behaved, the most robust, and the most active, were attacked and suffered equally as much as any patient I have seen with the epidemic cholera; out of a very considerable number of patients I only lost one man; the number I could not specify as I was ordered back to Jaulnah on duty about ten days after the appearance of the disease, and before the monthly returns were dispatched."

On referring to the returns of that corps it appeared, that in the month of June, 1814, ninety-nine cases of "bowel complaint" were entered, of which fourteen proved fatal; and about sixty cases of the same disease were admitted in the succeeding two months, of which however very few died. As these returns made no allusion to cholera, and as they were signed by Mr. Cruickshanks, a reference was made to him for information, respecting the preceding observations of Mr. Duncan, which has drawn forth the very valuable report, inserted at page 234, and bearing date the 17th June, 1823. It now appears, that a brigade of two Battalions of N. I. marched from Jaulnah on the 29th May, 1814, and that about the 10th or 11th of June a disease broke out in one of the Corps, which there can be no doubt was the spasmodic cholera. "*When taken into hospital,*" *Mr. Cruickshanks observes of the first cases he saw,* "*they exhibited all those symptoms now so well known, of persons labouring under the advanced and fatal stage of epidemic cholera; the skin cold, and covered with cold perspirations; the extremities shrivelled, cold, and damp; the eyes sunk, fixed, and glassy, and the pulse not to be felt. These persons all died, and I find, on referring to such notes as I have preserved, that influenced by consideration of the vascular collapse, and total absence of arterial pulsation, I had denominated the disease asphyxia.* Many sepoys were brought into hospital in circumstances approaching to those above detailed. Of them, in a considerable proportion, the disease terminated fatally. *Thus the cases which I first saw of this malady, in the aged among the camp followers, differed in no respect from the worst cases of that*

*affection since so well known under the name of spasmodic cholera.* That name however I did not adopt, neither in my public reports, nor in the private notes which I took at the time. In this I was chiefly influenced from considering the nature of the matter ejected by vomiting and by stool, which in cholera is said to consist of bile, but *which in these cases was aqueous or mucilaginous.* Besides it was evident that the diluent treatment, recommended in cholera, could never be applicable to such a disease as that with which I had to contend. I continued, therefore, to employ in my reports the term "bowel-complaint," both because I found it in the hospital books on joining the corps, and because, if it conveyed no very precise idea of the malady which it was meant to designate, it was at least an appellation whence no erroneous impressions could be derived."

This paper by Mr. Cruickshanks is of great importance, *in as much as it evinces that cholera did exist to an extent not hitherto suspected to have occurred at so recent a date; and also, that even under these circumstances, no trace of it is found in the public records, for unless we had been guided by the incidental remark of Mr. Duncan, made five years after the occurrence, and had most fortunately been able to refer to Mr. Cruickshanks, the medical returns of the corps never could have led to a knowledge of it.* Hence, as already observed, though cholera very rarely appears in the sick returns of former times, *it is by no means to be thence inferred that it did not then exist.*

But this paper is also peculiarly valuable, as shewing that the cholera assumed, on that occasion, one of those singular and unaccountable features which it has frequently manifested in the present times; for, after enumerating various striking atmospherical vicissitudes, change of food, and many other predisposing, remote, and exciting causes of disease, to which the Brigade had been exposed, Mr. Cruickshanks goes on to observe, "To none of these causes of disease which I have enumerated, did the natives themselves attribute the sickness and mortality which prevailed; and on considering that of two Battalions composing the Brigade, alike exposed to all those causes, *one only* suffered from the epidemic, *the hospital of the 5th N. I. exhibiting not a single case of analogous disease,* those adduced can only be regarded in the light of remote, or predisposing causes; while, something or other acting exclusively on one Battalion must be sought for as instrumental in exciting the malady." We shall have occasion hereafter to revert to this particular subject; at present it is mentioned as shewing that cholera did, even then, manifest one of the most curious of its features, namely, that of two bodies of men, apparently under similar circumstances, one shall be attacked by it, and the other shall escape.

[*Mr. Hay considers it to be endemic in Travancore.*] It would also seem, by the subjoined extracts of reports from Mr. Staff Surgeon Hay, that cholera, in a form nowise different from the spasmodic or epidemic, is endemial in the Travancore country, and that he regarded the disease which appeared there in October, 1818, to be this endemic, rather than the epidemic, whose approach from the northward he still contemplated. On the 19th November, 1818, Mr. Hay writes; "the spasmodic cholera I am happy to say abates; the last seven days not having afforded more than thirty-six cases at Quilon, and there has been no casualty here in that time; but the Vythians, who arrive from the country for instruction and medicines, report the deaths of almost all attacked." After acknowledging the receipt of some medical supplies he continues, "I trust to be able to make a noble stand against the *epidemic* when it arrives; what I have had to encounter recently I hold to be the *endemic* Veshoo-ugeka, or Neer-comben, if not of the Malabars, certainly of the Travancorians, which is perfectly familiar to all here; committing frequently great mischief, and sometimes (25 years since) desolating the country. Then, thousands are said to have died of it; the Vythians fled from it as a plague, and no one, who has not early succour from suitable medicines, is ever known to recover;" "the description of the Veshoo-ugeka tallies in every particular with that of the spasmodic cholera; and whether the epidemic reaches us or not, the country will have reason to be thankful for instruction and remedies they never might have had, unless the dangerous inroad of the epidemic had been apprehended. In May last at Trevanderan, the capital, one hundred lives were sacrificed to this Veshoo-ugeka (poisonous air); some of the servants of the palace were seen by Mr. Provan's Assistants, and saved, but the villagers around, having no assistance, died to a man." Again, on the 24th December, 1818, Mr. Hay writes; "The Neer-comben, which signifies gush of water by stool, the effect of the disease, and its synonyme Veshoo-ugeka, or poisonous air, its imputed cause, which are the vulgar and scientific designations of our present spasmodic cholera, has been very prevalent amongst the troops, their families, and followers. In Quilon I have treated upwards of 120 under the spasmodic cholera, and of the inhabitants a considerably greater number, with complete success in every case, where application was made within six hours; and hundreds have been saved by the use of the remedies I have distributed throughout the country. This shews unusually, for be it remembered that to the central parts of the Travancore coast, and parts *quite* adjacent, so far as my reports inform me, the *endemic* has been principally confined, and it is of this I speak; but the *epidemic* also now rapidly progresses southward, having already at Cochin yielded Mr. Mather some hundreds of patients, and at Aleppy about 30 per diem are taken ill; as it nears us, I become more apprehensive that the mortality will be great, for although medicines, with ample instructions, have been distributed to 140 Vythians and others in the country, yet from the experience I daily have of their general inattention, I much fear that when the day of visitation and trial arrives, the sick will be found too often left to their fate, altogether unassisted." Mr. Hay goes on to state, that in some villages where there was no medical aid, from three or four to ten people were dying daily of the *endemic;* and, talking of the zeal of the Vythians, he observes, "but when the same malady (spasmodic cholera) was *epidemic* here 34 years since, they ran from their charge, under the persuasion that the disease was contagious, for many died, and numbers in one family."

There can be no doubt, however, that the disease described here as an endemic was, in fact, the epidemic cholera of other parts; and no particular manifestation of it took place afterwards at Quilon in regular course from Cochin and Allepy, as seemed to have been expected by the Staff Surgeon, nor, indeed, until July and August following. The progress of cholera as an epidemic along the western coast, however, was much less regular than in other tracts, which may perhaps be attributed partly to the geographical peculiarities of that coast, and partly to the disease being in some degree endemic, which would not only accelerate the invasion and march of an epidemic of the same nature, but also render it difficult to fix the precise dates of its appearance.

[*Epidemic Attack of Cholera in Travancore, about* 1790, *and in the Ceded Districts about the same Time.*] Mr. Hay mentions in the first letter, that Cholera committed great ravages in the Travancore country "25 years since," and in the 2d letter that "it was epidemic 34 years since;" either of these dates, supposing that there was only one visitation meant, would prove the existence of cholera, epidemically, at a period considerably ulterior to 1787, and of course anterior to the instance in the 1st Battalion, 9th Regiment; and the whole communication shews that the disease is at no time of rare occurrence in that country. There is a very fatal form of disease also known in Travancore, called "the red eye sickness" by the natives, which is evidently a modification of cholera. Mr. Superintending Surgeon Duncan (page 110) also observes, "I find the old inhabitants of Bellary are acquainted with this disease, and inform me that it raged here about 30 years ago with great violence. This was succeeded by a famine, for want of inhabitants to cultivate the country."

Having, in the preceding desultory remarks, attempted to trace the existence of cholera in India from a very remote period, down to that of its epidemic appearance in 1817, sometimes coming as a pestilence upon the land, at others visiting only particular tracts; and having also attempted to shew grounds for inferring, that we are not acquainted with all the instances of its epidemic visitations, nor by any means aware of the extent of its occasional or sporadic appearances, it only remains to refer the reader to the valuable reports of the Bengal and Bombay Boards, for information respecting its late march through the respective territories of those presidencies; to Mr. Orton's separate work, for many interesting particulars of its appearance here; and to the narrative at the first page of this report, for an account of its progress through the Madras territories. This narrative has been compiled from official reports; and, as it is intended to exhibit the history of the disease, as a sort of memoir to the map which is prefixed, it has been as much as possible divested of all medical reasoning. It is not intended to enter into any discussion respecting the forms of cholera, or the diseases supposed to have been cholera, which may be thought to be described by European writers of ancient, and modern times, as having prevailed in Europe.

[*Nosological Remarks.*] Cholera has generally been classed by Nosologists under the head of Fluxus; but Cullen, though retaining the name, which he understands to signify "a flux of bile," and defining the disease to be so, or of "a bilious humour," places it in his class, Neuroses, and constitutes it a genus of the order, Spasmi. Dr. Good, in his late valuable work, the Study of Medicine, retains the generic term Cholera, which he justifies on the ground that the "bile is morbidly affected in its secretion, either in quantity or quality;" and he places it in the class Cæliaca, or diseases of the digestive function, and in the order Enterica, or diseases affecting the alimentary canal.

[*Names given to Cholera by the Hindoos.*] The quotations which have been given from Hindoo writings, shew, that cholera, or at least, two diseases resembling it, are also there classed, either under the head of nervous diseases, or of disorders of the digestive organs. The native practitioners know the disease by the name of Vishuchi, or Vishuchiki; but the people in general designate it only by two words, signifying in their respective languages, *vomiting and purging*. The term "Neer comben," mentioned by Mr. Hay, as being in use with the natives of Travancore to express the disease, does not appear to be known to the natives of the Coromandel coast. [*Mordixim.*] The word Mordixim, mentioned by several modern authorities, is incidentally introduced by Bontius in his description of the Hog stone, who says, that "it (the Hog stone) is infused in wine for the *Cholera* which the Islanders, (Malays) call Mordexi." Sonnerat has been accused of translating, or transforming this word into Mort de Chien; but independently of there being no such phrase in the French language, it is manifest, from the quotations already given, that Mort de Chien was in current use among our soldiers and sailors at the time Sonnerat wrote his book: he does not apply it indeed to cholera, but to an indigestion or cholic, in which sense it is in current use with the Portuguese at this day.

[*Generic Name.*] The generic term, cholera, being consecrated by universal, and almost immemorial use, it would not, perhaps, be proper to reject it, even could we propose another, demonstrably better; or prove satisfactorily, that bile, either in its quantity or quality, has no connexion whatever with the cause of the disease; but the specific terms may admit of some observations. We have had no other method in this country of distinguishing between the two forms of the disease, than by retaining the old, and, according to Dr. Good, the pleonastic appellation of *Cholera Morbus*, signifying that form wherein bile appears early, or from the first in the discharges from the alimentary canal, and in which the circulation is not remarkably depressed; and by applying the adjuncts *spasmodica* or *epidemica* to the second, or that form wherein bile, in common with the other glandular secretions, disappears, and where the pulse sinks, or ceases to be felt.

[*Specific names.*] The specific term, spasmodic, applied to the Indian cholera, has met with very serious opposition; for, if restricted to the affection of the muscles of voluntary motion, it implies a symptom of very minor importance, which in a great proportion of cases indeed, does not at all occur; and of which the existence in other parts of the system, cannot by any means be held as incontestibly proved. The term "cholera epidemica" is that which has been chiefly used of late, especially in official papers, and hitherto it has been sufficiently understood; but it is obviously adapted for temporary application only. It may therefore be allowable to substitute a term which, it will be attempted to shew, imports an unfailing diagnostic of this species of cholera; namely, the *sinking* or *arrest* of the circulation; and, accordingly, to call it cholera asphyxia, using the word asphyxia only in its restricted sense, that is the stoppage, or suppression of the *pulse*.

This proposed specific term, asphyxia, will, it is presumed, designate the disease unerringly; for, as far as our knowledge of it either from history or observation hitherto extends, there appears to be in all cases an evident tendency to a sinking of the circulation, an apparent arrest of it in the vessels of the extremities, if we may judge from the absence of pulse, and from the effect of venesection, in every instance where the complaint is not early cut short by art; and, especially, an arrest of it in every vessel accessible to the senses, in all fatal cases, at a period before death, comparatively more remote than is known in any other disease.

*(To be continued in the Appendix, page 609.)*

### APPENDIX.

### REPORT OF THE MADRAS MEDICAL BOARD ON EPIDEMIC CHOLERA.

*(Continued from Page 512.)*

The term asphyxia appears to have also occurred to Mr. Cruickshank, in 1814, as applicable to the disease since known as the spasmodic or epidemic cholera, though he did not propose to use it as an adjunctive, or specific term for cholera, but in a generic sense. The coincidence is still felt, however, to be confirmatory of the propriety of the appellation of cholera asphyxia, which was adopted in this report, long prior to the receipt of his communication bearing date the 24th July, 1823.

In the following remarks, accordingly, the term cholera biliosa will be used in speaking of the common form of the disease, or cholera morbus; and cholera asphyxia, or simply cholera, in speaking of that epidemic which forms the immediate subject of this report.

[*Description of Cholera.*] The symptoms of cholera asphyxia can hardly be better detailed than they are by several of the older authorities, whose descriptions of the disease have been quoted; or, than in many of the original papers which constitute the chief value of this work. The descriptions given in Mr. Jameson's, and Mr. Orton's publications are likewise of the highest value, and little more is left to be desired on that head than a few brief and supplementary observations.

*This most formidable disease does not appear to be attended by any premonitory symptoms which can be regarded as being at all peculiar to it; on the contrary, we may safely assert, that it is of sudden invasion:* for, though a slight nausea, a laxity of the bowels, and a general feeling of indisposition are often found to precede cholera, yet these symptoms are evidently common to many acute diseases; and they are especially frequent in this climate without being followed by any graver ailment. When such symptoms are found to precede cholera, they might with more truth be regarded as indicating merely a certain deranged state of the alimentary organs, a condition of the body which certainly predisposes a person to an attack of cholera.

The invasion of cholera generally takes place in the night, or towards morning. The patient is sick at stomach, he vomits its contents, and his bowels are at the same time evacuated. This evacuation is of a nature quite peculiar to the disease; the entire intestinal tube seems to be at once emptied of its fæcal or solid matters; and an indescribable, but most subduing feeling of exhaustion, sinking, and emptiness is produced. Faintness supervenes, the skin becomes cold, and there is frequently giddiness, and ringing in the ears. The powers of locomotion are generally soon arrested; spasmodic contractions, or twitchings of the muscles of the fingers and toes are felt; and these affections gradually extend along the limbs, to the trunk of the body. They partake both of the clonic and tonic spasm, but the clonic form chiefly prevails. The pulse, from the first, is small, weak, and accelerated; and, after a certain interval, but especially on the accession of spasms, or of severe vomiting, it sinks suddenly, so as to be speedily lost in all the external parts. The skin, which. from the commencement of the disease, is below the natural temperature, becomes colder and colder. It is very rarely dry; generally covered with a profuse cold sweat, or with a clammy moisture. In Europeans, it often partially assumes a livid hue; the whole surface appears collapsed, the lips become blue, the nails present a similar tint; and the skin of the feet and hands becomes much corrugated, and exhibits a sodden appearance. In this state, the skin is insensible, even to the action of chemical agents; yet the patient generally complains of oppressive heat on the surface, and wishes to throw off the bed-clothes. The eyes sink in their orbits, which are surrounded by a livid circle: the corneæ become flaccid, the conjunctiva is frequently suffused with blood: the features of the face collapse; and the whole countenance assumes a cadaverous aspect, strikingly characteristic of the disease. There is, almost always, urgent thirst and desire for cold drinks, although the mouth be not usually parched. The tongue is moist, whitish, and cold. A distressing sense of pain and of burning heat at the epigastrium are common. Little or no urine, bile, or saliva, is secreted. The voice becomes feeble, hollow, and unnatural. The respiration is oppressed, generally slow; and the breath is deficient in heat. During the progress of these symptoms, the alimentary canal is very variously affected. After the first discharges by vomiting and purging, however severe these symptoms may be, the matter evacuated is always watery, and in a great proportion of cases, it is colourless, inodorous, and often homogeneous. In some, it is turbid, resembling muddy water; in others, it is of a yellowish or greenish hue. A very common appearance is that, which has been emphatically called the "conjee stools," an appearance produced by numerous mucous flakes floating in the watery or serous part of the evacuation. The discharges from the stomach, and those from the bowels, do not appear to differ, except in the former being mixed with the *ingesta*. Neither the vomiting nor the purging are symptoms of long continuance. They are either obviated by art, or the body becomes unable to perform these violent actions; and they, together with the spasms, generally disappear a considerable time before death. If blood be drawn, it is always dark, or almost black, very thick, ropy and generally of slow and difficult effusion. Towards the close of the attack, jactation comes on, with evident internal anxiety and distress: and death takes place, often in ten or twelve, generally within eighteen or twenty, hours from the commencement of the attack.

During all this mortal struggle and commotion in the body, the mind remains clear, and its functions undisturbed almost to the last moment of existence. The patient, though sunk and overwhelmed, listless, averse to speak, and impatient of disturbance, still retains the power of thinking, and of expressing his thoughts, as long as his organs are obedient to his will. Such is the most ordinary course of cholera asphyxia, when its tendency to death is not checked by art.

A favourable issue is denoted, by a rising of the pulse, a return of heat to the surface, inclination to natural sleep, and a diminution, or cessation of vomiting, purging, and spasms; these indications being succeeded, after an interval, by the re-appearance of fæcal matter in the stools, of bile, of urine, and of saliva.

[*Varieties in the general Features of Cholera.*] Cholera, like other diseases, has presented considerable variety of symptoms: but, before we proceed to notice its more striking varieties, it is necessary to advert to one feature, which, though not altogether unobserved in other epidemics, may still perhaps be regarded as especially distinguishing cholera; namely, that these varieties are not observable so much in *individual* cases, as, in what may be termed, *local epidemic visitations*. Thus, when the disease appears epidemically in a town, or district, or in the lines of a corps, or the camp of a marching regiment, it may on one occasion be distinguished, throughout, by the absence of vomiting, and the prevalence of purging; on another, by the excess of vomiting, and, though more rarely, by the absence of purging. Spasm may be generally present in one instance of invasion; in another it may not be distinguishable. A frequent variety, the worst of all, is that which is noted for the very slight commotion in the system; in which there is no vomiting; hardly any purging; perhaps only one or two loose stools; no perceptible spasm; no pain of any kind; a mortal coldness, with arrest of the circulation comes on from the beginning, and the patient dies without a struggle. This has frequently manifested itself as the prevailing type; and almost all die who are attacked by it: but fortunately it has not usually lasted long; the disease either disappearing, or assuming during its further progress, a milder or less formidable character.

It would be highly important in a pathological view, could we trace these leading distinctions in the disease, to any particular state of the weather, to any local peculiarity, or to any circumstance affecting the food, shelter, or occupation of the people, who may be the subjects of it; but it must be confessed, that this is far from being practicable. It appears, on the contrary to be established, that under circumstances apparently similar in all respects, these modifications of the disease have been found equally to prevail. On the other hand, it may be assumed, that a person, in proportion to the vigour of his constitution and to the unimpaired state of his health, is less liable to be affected with the low form of the disease.

[*Vomiting.*] Vomiting is a prominent symptom of cholera: but there are numerous instances on record where it has been entirely absent. In certain epidemic visitations even, scarcely an individual case has manifested this symptom. In some cases the stomach appears to be freely and perfectly emptied; prodigious quantities of watery fluid being ejected, occasionally with great force. This fluid sometimes resembles what is discharged in pyrosis; at other times it is glairy and ropy. In other cases, the stomach seems to have lost the power of freely ejecting its contents: there is an ineffectual straining to vomit, and a spouting up of any fluid which is swallowed, as if by an effort of the lower part of the œsophagus, rather than of the stomach itself. When full vomiting in these cases has been effected by medicine, relief follows; not, however, in all probability, by the mere evacuation of the gastric contents, but as a consequence of that change in the condition of the patient, which must necessarily be established before the stomach can resume the action of vomiting. Vomiting is sometimes altogether absent; or, if it has been present, soon ceases, from an atonic state of the stomach, under which that organ receives and retains whatever may be poured into it, as if it were really a dead substance. This is a most alarming state, in comparison with which, the utmost irritability, or almost any other imaginable condition of the part, may be held to be of little danger.

It is not always easy or possible to ascertain what substance imparts the greenish or yellowish hues to the fluids ejected by vomiting: but it is perhaps too readily admitted, that these colours are owing to the admixture of bile. A regular series of experiments, regarding the effects of chemical agents on the gastric and intestinal secretions, and on the matters discharged in cholera, and in other diseases, is certainly a desideratum. The Medical Board have addressed a circular letter to several of their officers on this subject, which it is hoped will ultimately be the means of drawing forth some precise information.*

Supposing, however, that either the yellow or green hue of the matter vomited in cholera, indicates the presence of bile, it is undoubtedly of rare occurrence, especially during the acute stage of the disease. It would appear, nevertheless, that apparently bilious matters have been vomited, particularly at the beginning, and towards the favorable termination of the disease, and even in cases, which have ended fatally. The mere presence of bile in the discharges cannot, therefore, be held as decisive against the disease being the cholera, of which we are treating. Worms, especially the lumbricus teres, have been very generally ejected by vomiting: and several medical officers have noticed, that this symptom has even indicated a less dangerous form of the disease. If there be any truth in the observation, it is probably referrible to the *free* action of vomiting, which brings up these animals; this being in itself a favourable symptom.

[*Purging.*] Purging is a more constant symptom of cholera than vomiting: and in a majority of cases, it is the first in the order of occurrence; but, being a less striking deviation from a state of health than vomiting, which instantly arrests the attention, it has usually been treated of in succession to it. This symptom has very rarely been altogether absent: but there seems no reason to doubt, that this is sometimes the case. Its absence appears indeed to denote a peculiar degree of malignancy in the attack. The accounts given by the patients, however, in respect to their alvine evacuations, are not to be impli-

---

* "On mixing 20 grains of calomel with an ounce of ox bile, which was previously of a "grass green colour," the bile assumed the hue of "pea green." This hue was rendered more intense by the application of heat, and took on a tinge of yellow. After standing 24 hours, the mixture was of "a dark or sap green." The calomel appeared at the bottom like "blue ointment:" and was "unctuous" to the feel. On mixing 40 grains of calomel with an ounce of ox bile, previously of a "light brown colour with a shade of green," the mixture assumed the colour of "ochre." After standing it assumed a "grass green colour." The calomel was discoloured in a less degree than in the former experiment. On mixing 15 grains of calomel with 6 drachms of sheep's bile, which was of a "dark brown colour with a shade of green," the mixture put on a "light pea green with a tinge of yellow." On subsidence of the calomel, which was apparently unchanged, the bile appeared of a "rich dark green colour." On mixing 40 grains calomel with two drachms of goat's bile, which was of a "dark green colour," no change of colour took place.

On mixing 4 drachms of ox bile of a "light brown colour," with an equal quantity of liquor ammoniæ, the mixture assumed the colour of madeira wine. Equal parts of sheep's bile and liquor ammoniæ being mixed, the fluid became of a light yellow colour. The bile was previously of a deep yellow.

"On mixing equal parts of sulphuric æther and ox bile, which was previously of a pea green colour," the fluids did not unite. The æther got a "yellow tint: and the bile became of a yellowish hue."

These observations have been kindly communicated by Mr. M'Farland, Assist. Surg.

citly believed. Their attention is not always drawn to the nature of the discharge: and they are apt to convey very erroneous notions on the subject to the medical attendant. In cases, where little or no purging has taken place during life, the intestines have yet been found, after death, to be filled with the conjee like matter, as if they had wanted energy to throw it off, or, as if a stricture had been formed on the lower portions of the gut. The intestinal canal appears to be subject indeed to the same influences, and its contents appear to vary, as has been stated to be the case with the stomach, with this exception, that it seems always to have the power of emptying itself of its *natural* contents at the commencement, or during the progress of the disorder. This inference is drawn from the accounts of dissections; for we find no instance recorded of fæces remaining, unless in very protracted cases, when the primary disease has been overcome. The dejections are sometimes made without effort or uneasiness; at others, they are thrown out with great force, which has been compared to the squirt of a syringe. They also sometimes take place simultaneously, with vomiting, spasm, and stoppage of the pulse, as if all these affections originated, at the instant, from one common cause. There is seldom much griping or tenesmus, although the calls are very sudden, and are irresistible. Pain on pressure of the abdomen is only occasionally noticed. In advanced stages of the disease, purging generally ceases: but in many cases, a flow of watery fluid from the rectum, takes place on any change of position. The matters evacuated, after the first emptying of the bowels, have been occasionally observed to be greenish, or yellowish, turbid, of a frothy appearance like yeast, and sometimes bloody. In some cases they are inodorous, in others they have a rank fleshy smell. In one fatal case, pure bile, it is said, was discharged. Perhaps much of this variety may depend on the previous state of the large intestine, especially in Europeans, who so generally labour under a morbid condition of that organ: but, by far the most common appearance is that of pure serum, so thin and colourless as not to leave a stain on the patient's linen. The next in order of frequency is the conjee like fluid; the mucus is at times so thoroughly mixed, however, with serum, as to give the whole the appearance of milk or chyle. The evacuations have also been observed to resemble soogee in colour and consistence: and these cases were mild. Worms are very commonly discharged by stool. The reappearance of fæcal matter, especially if tinged with bile, seldom, perhaps never, takes place till the disease has been subdued. The quantity of the clear watery fluid, which is sometimes discharged, is exceedingly great; and, were it uniform, it might afford us an easy solution of the debility, thirst, thickness of the blood, and other symptoms: but it is unquestionable, that the most fatal and rapid cases are by no means those, which are distinguished by excessive discharges. *We have innumerable instances, on the contrary, of death ensuing after one or two watery stools, without the development of any other symptom affecting the natural functions. Even collapse has come on, before any evacuation by stool had taken place.**

[*Of the Animal Functions.*] Though the animal functions necessarily partake of that disorder of the vital and natural functions, which very strikingly characterises cholera, yet this participation is not so immediate as we might, à priori, be led to expect.

The undisturbed state of the mind has been the subject of general remark: but it cannot be matter of surprise, should some exceptions occur, from a fortuitous morbid affection of the brain following a state of sanguineous congestion. There is reason to believe, that the simple congestion observed in cholera has not been the cause of the coma, or insensibility, which have been remarked: and, when we recollect, that almost all practitioners advert to the great reluctance of the patient to be aroused, we must admit, that cases of imputed coma may have often been referrible to this condition, which have yet been reported as arising from physical disability. Instances are not wanting of patients being able to walk, and to perform many of their usual avocations, even after the circulation has been so much arrested, that the pulse has not been discernible at the wrist. Much seems to depend on the constitutional strength, and firmness of mind in the patient, and on the form, in which the disease has made its attack. The cases here alluded to are those chiefly, in which it has begun by an insidious watery purging: and many lives have been lost in consequence of the patients, under these fallacious appearances, not taking timely alarm, and applying for aid. In other cases again, the animal functions appear to have been early impaired, and the prostration of strength to have preceded most of the other symptoms.

---

* See Dr. Clarke's Description of the Smyrna Disease.—ED.

[*Spasm.*] Spasm has been held to be so essential a feature of that species of cholera, of which we are treating, as to confer on it a specific name. In so far, however, as relates to the muscles of voluntary motion, and it is that description of spasm only, which we mean here to treat, no symptom is more frequently wanting. Spasms of the muscles chiefly accompany those cases, in which there is a sensible and violent commotion in the system. Hence they are more frequently found in European than in native patients; and in the robust of either, than in the weakly. In the low, and most dangerous, form of cholera, whether in European or native cases, spasm is generally wanting, or is present in a very slight degree. The muscles most commonly affected are those of the toes and feet, and calves of the leg; next to them, the corresponding muscles of the superior extremities; then those of the thighs and arms; and, lastly, those of the trunk, producing various distressing sensations to the patient. Amongst these, hiccup is not unfrequent, but it has been observed that this symptom, in cholera, is not at all indicative of danger. The muscles of the eye-balls have not been observed, to be affected with spasm, unless the sinking of these organs in their orbits may be considered to be an effect of it. The reports make frequent mention of a remarkable, permanent contraction of the muscles of the abdomen, by which the belly is drawn towards the spine. The spasms attending cholera are of a mixed nature, not strictly clonic; the relaxations being less prompt and frequent than in epilepsy or convulsion; and seldom durable as in tetanus. The contractions of the muscles are invariably attended with pain, and some Medical Officers have observed, that a degree of spasmodic stiffness has continued for several days afterwards. It has also been remarked that spasmodic twitchings of the muscles have taken place after death, and have continued for a considerable time. In one case where a man had been paralytic in his limbs, with a total numbness of them, they were severely affected with spasms, and became exquisitely sensible. It is pretty evident, that there either has been an inaccuracy in the description of spasm occurring in cases of cholera, or a sensation differing from that of spasm has been confounded with it; for, by the descriptions, we would be led to suppose that the spasms begin, and are felt, *in* the toes and fingers, which cannot be the case. As the extreme muscles however are generally first seized with spasm it is probable that the small fleshy bundles in the palms of the hands, and soles of the feet *are* affected; but there seems reason farther to conclude, that pain *is* really felt *in* the fingers and toes, and that it is referrible to a sort of nervous twinge or tic doloreux in these parts, distinct from spasm, which is not uncommon in other disordered states of the digestive organs.

[*Collapse.*] Of all the symptoms of cholera, none is so invariably present, none indeed so truly essential and diagnostic, as the immediate sinking of the circulation. It must nevertheless be admitted, that, where instant remedial measures have been successfully practised, this symptom may not have developed itself; and that there are even cases, where an excited vascular action has been observed to accompany the first movements of the system in cholera. Some intelligent practitioners have entertained doubts whether such cases belong indeed to this disease; and there seems reason to imagine that those inflammatory affections with spasm, known in this country, and alluded to in several reports, may, in some instances, have been mistaken for it. It is farther to be remembered that these are precisely the cases, which yield most certainly and readily to our remedial means: and it consequently follows, that a medical man can seldom have the opportunity of observing, whether or not, this form of cholera will degenerate into the low stage. There is, however, direct evidence in support of the fact, that they have so degenerated, and gone on to a fatal termination. In the case of soldiers too, in whom such symptoms have chiefly appeared, we must make some account of the quantity of spirits usually drank by them at the commencement of the disease, producing an effect on the circulation. The period, at which a marked diminution of vascular action takes place, is somewhat various. The pulse sometimes keeps up tolerably for several hours, though very rarely. It more generally becomes small and accelerated at an early stage; and, on the accession of spasm, or vomiting, suddenly ceases to be distinguishable in the extremities. The length of time, during which a patient will sometimes live in a pulseless state is extraordinary. Dr. Kellett relates a case, where the pulse was gone within three hours from the attack; yet the man lived in that state, from the 3d October at 4, P.M., to the 6th at 2, P.M. On the cessation of the spasms or vomiting, and sometimes, apparently, from the exhibition of remedies, the pulse will return to the extremities for a short time, and again it will cease. The superficial veins and arteries are not always collapsed, even when the pulse has ceased. If these vessels be opened in this condition, the contained blood flows out; their walls then collapse, and no more blood can be extracted. There is no authenticated fatal instance of cholera on record, where the circulation has not been arrested, in the extremities at least, long before death took place. The only apparent exception to this conclusion would not have been deemed deserving of particular attention, were it not, that in the faithfulness of record, which it is hoped distinguishes this report, nothing, purporting to be a medical fact or observation, is omitted. The case is this. "Scarcely any disease occurred on the march, with the exception of a few cases of cholera; of these, a havildar and sepoy died, and several followers, who seldom were reported in time to receive any aid. One of the last class, a fine stout young man, a bullock-driver, was brought to the hospital almost in the last agony; I mention this case from the peculiarity of the patient's skin being hot and dry, and his pulse being about 120, full and strong, until the last moment, circumstances I had never seen before; while from the peculiar appearance of the eyes, and the collapsed features, together with the description given of the attack, and progress of the disease left me no room to doubt its being genuine. 1st July, 1821." The writer of the preceding extract notices the case for its singularity; and, in judging of its identity with cholera asphyxia, we must allow due weight to the circumstance of his attention being thus excited. He acknowledges, however, that the patient was brought to him in the last agony, and draws his conclusions from the reports of the manner of attack and progress of the disease, and from the appearance of the features of the face. We are left to conjecture as to the former of his grounds of belief; and shall, therefore, only hint at the possibility of coup de soleil having been mistaken, *on description*, for cholera: but the sudden collapse of the countenance, which takes place in cholera, seems hardly reconcileable with any condition of body, in which the skin is dry and warm, and the *pulse full and strong!* That peculiar state of countenance is manifestly the result of the retrocession of the blood from the surface; and it is quite distinct from the wasting of the solid parts from disease, or inanition. That a case of cholera may terminate in death, and the pulse remain *at* 120, *full and strong*, to the last moment, is not physically impossible, although at present standing single on the records: but, when supported by hearsay evidence, and by an observation, which, it has been attempted to shew, is not tenable, we may be allowed to regard it as one of those *facts* alluded to in the beginning; and, being palpably at variance with general experience, it is to be weighed with much caution and circumspection, if not totally rejected.

[*Thirst and sense of heat in the epigastrium.*] Thirst, and a sense of heat, or burning, in the region of the stomach, are generally connected together, and form very prominent and constant symptoms of cholera: yet, not only in individuals, but even in epidemic invasions, these symptoms have often been altogether wanting. Even when they are present in the highest degree, the mouth is not often parched, nor the tongue often dry; on the contrary, there seems in general no want of moisture; and while, as Mr. Jameson observes, "all is burning within," these surfaces are cold and blanched. At times, however, the mouth is parched, and the tongue dry and furred: but practitioners seem doubtful, whether any practical inference is thence to be drawn. What would be the state of these parts, if calomel, ardent spirits, laudanum, and spices were as largely employed in health, or in many common diseases, as in cholera, with as scanty a use of diluents? Might we not perhaps say, that a parched mouth and furred tongue, following the exhibition of such remedies in cholera, is rather favourable than otherwise, as indicating an action counter to that of the disease? When thirst *is* present, it seems to subdue all other feelings; and the ignorant soldier, as well as the medical man, who firmly believes that cold water is almost certain death, alike eagerly seek and swallow it. Two melancholy instances are recited, where Medical Officers have exerted their last and utmost efforts to reach, unperceived, even the water of the bathing tub; so intolerable are the pangs of this cruel thirst.

[*State of the Skin.*] The state of the skin in cholera is, in general, what we might expect to find it in patients labouring under such affections of the alimentary canal, and with the subdued circulation, which takes place in that disease. It is cold, generally clammy, and often covered with profuse cold sweats. Nevertheless, varieties occur in this, as in the other symptoms of cholera. The skin is sometimes observed to be dry, though cold; and sometimes of natural, nay, in some rare instances, of preternatural warmth. An increase of temperature has been repeatedly observed to take place just before death; but the development of heat appears to be confined then to the trunk and head; and, in almost all cases, this *partial* development of heat is found to be a fatal symptom. It is entirely unconnected with any restoration of the energy of the arterial system, or any improvement in the function of respiration. The heat, in such instances, has been observed to continue considerable for some hours after death.

The sensation imparted by touching the skin of a person ill with cholera is very peculiar, and reminds one of that imparted by a dead body. The skin, when much collapsed, becomes insensible, even to the action of chemical agents; and hence the usual vesicatories fail in producing any effect. The application of mineral acids, and of boiling water, in this condition of the skin, produces little or no effect, and some patients are said not to have been sensible of the operation.

The action of mineral acids on the skin is not, however, vesication, but rather that of a cautery; the cuticle, and the extremities of the subjacent vessels, appearing to be destroyed by them. It has been said, that vesication could not be produced in some stages of cholera, because the production of serum was, in common with the glandular secretions, arrested; but, when we reflect on the readiness, with which serous fluids are poured out in that disease, we shall be rather disposed to refer the failure in the action of vesicatories, even of hot water, to the diminution or destruction of the nervous energy of the skin. It is certain, that in a body *but just dead*, the application of boiling water will vesicate readily; and, if the accuracy of the observation respecting its non-vesicating power in advanced stages of cholera be established, we must infer, that there is less vitality in the skin in such cases, the patient being still alive, than in that of a body *recently* dead of some other disease.

At a very early stage in cholera leeches can procure little or no blood from the skin. This fact is noticed by some in another sense, as if these animals turned in abhorrence from the skin of a person ill with cholera. When the sweat is thin, it is usually poured out, in large quantity, from the whole surface of the body; but, when thick or clammy, it is more partial, and generally confined to the trunk and head. The action of the vapour, and hot baths, seems unquestionably to increase the exudation, or secretion from the skin; and the application of dry heat, as the natural temperature of the skin augments, appears to restrain these discharges; circumstances not very compatible with the supposition of a state of spasm of the vessels of the skin. The perspiration or moisture is often free from odour; at other times it has a fetid, sour, or earthy smell, which has been said to be peculiarly disagreeable, and to "hang long about the nostrils" of the bye-stander.

[*Countenance.*] That remarkable shrinking of the features of the face, which has acquired the emphatic term of the "true cholera countenance," appears in every case, not quickly cut short by medicine: but the degree, in which this symptom may be present, will be differently estimated, according to the natural contour of the patient's features. This expression of countenance, which conveys too truly that of death itself, cannot be mistaken; and, by an attentive observation it will be perceived, that a similar shrinking takes place throughout the limbs and all projecting parts of the body. The eyes not only become dim, and the corneæ flaccid, but there appears to be an actual formation of a substance like a film, or membrane, in many cases: shewing, that this species of surface still possesses secreting powers. The abdomen has sometimes been observed to be tumid, but more frequently drawn towards the spine. The general apparent reduction of bulk, cannot however be considered as proportionate to the volume of fluids thrown out; nor, indeed, to depend essentially on that circumstance, as it occurs equally under the most moderate discharges.

[*Respiration.*] Respiration is not usually interrupted in the early stages of cholera, unless from a peculiarity in the mode of attack, under which spasm seizes the muscles subservient to that function. In many cases terminating in death, respiration has gone on in its mechanical part with little or no interruption, except that it becomes slower and slower; and an instance has been recorded, where this function was performed only seven times in the minute. Numerous cases on the other hand are noticed, especially in Europeans, where the interruption of respiration was most distressing, and could only be compared to the most violent attacks of asthma. Although the breath is stated in many of the reports, to have been deficient in heat, it is not clear that this was a general symptom: nor is it understood, that this coldness was more particularly observed in cases of difficult and laborious respiration, than in those, where this function seemed to be, at least mechanically, performed without interruption.

[*Jactation.*] With respect to restlessness, or jactation, it is more common with Europeans than with natives. In cases of such sudden and dangerous illness, we must make some allowance for moral, as well as physical disquietude: and it is certain, that in very many cases, death approaches, while the patient lies in the most complete tranquillity. When much restlessness prevails, it is probably connected with some great oppression of particular organs; and, though the absence of this symptom is not, in itself, to be depended upon as affording grounds for a favourable prognosis, its presence is always highly alarming. The voice, in general, partakes of the debility prevailing in other functions, and is usually noticed as being feeble, often almost inaudible. Yet instances are not wanting, where the voice has continued of natural strength almost to the last moment.

[*Functions of the Sensorium.*] In a disease so highly congestive as cholera, where vertigo, deafness, and ringing in the ears, often prevail, and where very large quantities of opium and intoxicating matters have been swallowed, it is truly surprising, that the functions of the sensorium are so very rarely disturbed. It seems probable, that it is in many instances from an inaccuracy of language, that coma has been represented as a symptom of cholera: for we find that patients, who have just been represented to be in a *comatose* state, can, with more or less facility be roused from it; and, though he cannot overcome that retirement within himself, which constitutes so remarkable a feature of the disease, he will yet evince, by the clearness and precision of his answers, that his intellect is not destroyed. The same appearance takes place in tetanus, hydrophobia, and other diseases referred to the class of neuroses. This circumstance shews their affinity with each other, and is calculated to make us pause in receiving doctrines as true, which impute such disorders to depraved functions of the nerves, whose origin, the sensorium commune, nevertheless, remains comparatively undisturbed. Coma must, however, be admitted occasionally to occur, especially towards the termination of the case, when it is fatal: but delirium has seldom or never been observed, unless as a sequelæ of cholera, when other and foreign morbid actions have been established. That degree of incoherence, which has accompanied the excessive spasmodic affections of the muscles, or which has followed the free use of opium and spirits, is not considered as an exception to this remark.

Syncope is not a common symptom in cholera, and when it has occurred, unless after venesection, it has generally been on the invasion of the disease. During the progress of this disorder, when the nervous energy seems to be almost annihilated, and the functions of the heart and arteries to be abolished, this symptom is yet very rarely observed! Deafness has been remarked, in some instances, to have been completely established, before any other symptom of the disease had developed itself; the patient continuing, for a time, to pursue his ordinary employments.

[*Recovery.*] When medical aid is early administered, and when the constitution is otherwise healthy, *the recovery from an attack of cholera is so wonderfully rapid, as perhaps to be decisive of the disease being essentially unconnected with any organic lesion.* In natives of this country especially, in whom there is ordinarily very little tendency to inflammatory action, the recovery from cholera is generally so speedy and perfect, that it can only be compared to recovery from syncope, cholic, and diseases of a similar nature: but, in Europeans, in whom there is a much greater tendency to inflammation, and to determinations to some of the viscera, the recovery from cholera is by no means so sudden or so perfect. On the contrary, it is too often involved with affections as various as the diseases of these viscera are known to be in this climate. The most frequent of the sequelæ of cholera are affections of the intestines, of the brain, of the liver, and of the stomach. When cholera, however, is of long continuance, and when the *congestion* appears to have been thoroughly established, few, either Europeans, or natives who outlive the attack, are restored to health without considerable difficulty.

It has been already remarked, that recovery from an attack of cholera is indicated by the return of the heat to the surface of the body, and a rising of the pulse. A deceitful calm, however, sometimes attends these favourable appearances, which too often mocks our hopes and expectations. When the disease is characterised by violent morbid actions, the diminution or cessation of these, however sudden, may generally be regarded as the usual mode, in which nature conducts the patient to recovery: but, in what may be termed *negative* symptoms, the steps to recovery are extremely dark and obscure: and the evolution of natural heat, and arterial action have occasionally been noticed as amongst the last of the functions, which are restored. Patients have been observed to remain for one, two, and even three days in a state of the greatest collapse, and yet, contrary to all expectation, have recovered.

[*Urine.*] In cholera the secretion of urine, like all the other natural secretions, appears to be very generally suspended. This, indeed, has been considered so much a matter of course, that practitioners have very frequently not noticed it in their reports: but, wherever the secretion has appeared to be going on, the circumstance is particularly mentioned. When cholera first appeared, attempts were often made to relieve the patient by the catheter, under the supposition that the absence of urine was owing to *suppression*. When this secretion is not suspended during an attack of cholera, the urine is almost always limpid and clear, though in very small quantity; a curious phenomenon, considering the probable state of the blood under such circumstances: for we may be permitted to infer from all the symptoms, that the blood is not only deprived of much of its serous or aqueous parts, by the profuse discharges which usually take place, but that the elements of all, or most, of the other natural secretions, are retained in it. We might, therefore, naturally have expected, that, if urine were secreted at all, it would possess some striking deviation from its natural appearance. Admitting that the blood is not freed from the elements of the secretions, which usually take place in health, what effect may their presence be supposed to have in producing some of the symptoms of the disease?

It has been remarked, that the cases, in which urine appeared to be secreted, were not less dangerous than those, where this secretion was entirely suspended: but it is much more generally observed, that the appearance of urine, especially when this is the result of *restored* secretion, is always a most favorable omen. In many cases the secretion of urine has not been restored, before a period of 50 hours had elapsed from the commencement of the attack: and it has even been reported, that during a local prevalence of cholera, the secretion of urine has been, in some individuals, entirely suppressed, although no other derangement of the health took place. Instances of this kind were generally observed during great heats, and under much fatigue.

[*State of the Blood.*] No symptoms of cholera are so uniform in their appearance and progress, as those connected with the blood, and its circulation. Although the reports, in general, afforded ample reference to this point, it still appeared to the Medical Board to be one of such importance in the pathology of the disease, that a circular letter was addressed to about thirty Medical Officers, who were supposed, from their experience in the treatment of it, to be best qualified to afford information. Attention was especially directed to the following considerations: first the influence which the state of the blood, in those affected with cholera, might be supposed to have in producing some of the symptoms: second, the colour of the blood abstracted from a vein in a person affected with cholera: third, the colour of the blood after a certain quantity had been taken, and, the effect, which any alteration of colour might have on the condition of the patient: fourth, if arteriotomy had been practised, the colour of the arterial blood in cholera: and lastly, the period, from the first attack of the disease, at which blood was abstracted. It is established by the replies to this letter, as well as by an immense mass of concurrent evidence, that the blood of persons affected with cholera, is of an unnaturally dark colour and thick consistence. These appearances are very uniformly expressed by the terms, dark, black, tarry, in regard to colour; and by thick, ropy, syrupy, semicoagulated, in respect to its consistence. The change in the condition of the blood is likewise fully proved to be in the ratio of the duration of the disease; the blood, at the commencement seeming to be nearly, or altogether natural, and more or less rapidly assuming a morbid state as the disease advances. Some very rare cases are recorded where, however, this morbid state of the blood was not observable, although the disease had been for some time established: and instances have occurred, where the blood flowed readily, sometimes little altered, where, nevertheless, death ultimately ensued. The abstraction of blood has been found by all practitioners to be very difficult and uncertain; and the uncertainty has been variously imputed to the feebleness of the circulation, to the thick consistence of the blood, and to the combined operation of these causes. The blood drawn from patients, suffering under cholera, is stated to be generally very destitute of serum, never to exhibit the appearance of buff, and to be generally disposed to coagulate quickly. Several instances, however, have occurred, where the coagulation was slow, and imperfect. A great majority of the reports state unequivocally, that, after a certain quantity of dark and thick blood has been abstracted from a patient under cholera, it is usual for its colour to become lighter, its consistence to become less thick, and for the circulation to revive: such appearances always affording grounds for a proportionably favorable prognosis. In many instances, however, no such changes have been observed to accompany the operation of bleeding, while yet the result was favorable. The blood is generally found to be less changed in appearance, in those cases of cholera, which are ushered in with symptoms of excitement, than where the collapsed state of the system has occurred at an early period. The blood has been occasionally found, on dissection, to be of as dark a colour in the *left*, as in the *right* side of the heart; affording reason to believe that in the whole arterial system it was equally changed. The temporal artery having been frequently opened, the blood was found to be dark, and thick, like the venous blood: but it would appear, that this operation has not been performed in general, until the attempts to procure blood from the brachial or jugular veins had failed: little or no blood could be obtained, the artery merely emptying itself in a languid stream, not in a jet, and then collapsing. An instance is stated where the surgeon, despairing of other means, cut down upon the brachial artery, but so completely had the circulation failed, that no blood flowed. When reaction has been established, the blood occasionally shews the buffy coat.

It would have been highly interesting, if, in those cases of cholera, which were distinguished by the freedom of the mechanical part of the process of respiration, and by the absence of great alvine or cuticular watery discharges, the colour and consistence of the blood had, in a greater number of instances, been ascertained. The evidence on these points, however, must be acknowledged to be defective. Amongst natives, respiration is pretty generally free until the very last stage; and the colour and consistence of the blood, in the instances where venesection has been performed, has been very uniformly stated to be dark, whether excessive discharges prevailed or not. It may thence be allowable to conclude, without any farther particular evidence, that though the passage of the blood through the lungs has been free, its natural change is interrupted by cholera. The coldness of the body in cholera, not only on the general surface, but in the axilla, as ascertained by the thermometer, might also warrant us in concluding, that the *temperature* of the blood is under the natural standard: but the inference is not sufficiently established by accurate observation. With respect to the watery discharges, we are not always sure that during life the stomach or intestines have been emptied. These organs have sometimes been found, after death, to be filled with fluid, though no purging, or vomiting had been observed. Although, therefore, the thick consistence of the blood would seem to find a ready explanation in the circumstance of the profuse excrementitious discharges robbing it of its natural serum: and although the general tenor of the reports would seem to warrant the conclusion, it must yet be confessed there is reason to believe, that the blood has been found occasionally to present these appearances, when no such discharges had taken place.

[*The terminations of Cholera.*] The terminations of cholera will readily be apprehended from the observations, which have already been made. It is the declared opinion of many of the practitioners, who have had to cope with the disease, that its tendency to death is so great as never to be counteracted by the unaided efforts of nature. The same opinion is no less evidently implied by the observations of all, that the delay of but a few hours places the patient beyond the reach of art: for hours are, in this disease as days in any other. There are not wanting men, however, who, either from an affectation of singularity, or from the melancholy results of their own practice are said to doubt the power of medicine in the cure of cholera, and to ascribe the recoveries, which they have witnessed, to the natural, unaided powers of the constitution. Such feelings are too apt indeed to arise in the mind, when the sad experience of some malignant epidemic visitation, or a succession of intractable cases, comes to be contrasted with the more successful efforts of others, who have had a less formidable enemy to combat: but they are especially apt to arise on hearing of the reputed cures made by the aid of native establishments. It is obviously very difficult to arrive at the means of making a true estimate on this subject. Where the aid of the European practitioner has been applied, the mortality has still undoubtedly, been extremely great: and the accounts of native doctors cannot be at all relied on, either for their veracity, or their qualifications to discriminate the disease. Such of the reports of the native revenue servants as have come under the observation of the Medical Board, all tend to prove that by far the greater proportion of people, who suffered attacks of cholera, and had no efficient aid, died. The Ameen of Ganjam writes thus; "The people, who get the cholera morbus never recover: death to them is certain." The Resident at Hydrabad states, that he feared every case treated by the natives proved fatal. The family of a wealthy Nair in Travancore, consisting of 19 people, were all, save one, cut off in a few hours. Another family of five all died. Mr. Searle, at Mannatoddy, states, that of 28 villagers attacked with cholera, 26 died; the other two recovered by his assistance. Death may, therefore, be said to be the ordinary termination of cholera; and there is, in truth, very little variety in the course, which the disease pursues towards it. This has already been described as consisting in a general suspension of the natural, and a gradual cessation of the vital functions, rather than in the establishment of morbid actions. Cases have been remarked, where the vital functions have been more suddenly overcome, and where death took place before the usual development of the symptoms; others, where life is extinguished in some sudden convulsive commotion of the system; and some, as if from apoplexy. Fatal terminations likewise occur from topical inflammations supervening; as, gastritis, enteritis, or hepatitis.—The intestinal canal seems especially obnoxious to the effects of cholera, numbers of those attacked with it, having subsequently been seized with dysentery. The favorable issue of cholera certainly resembles that of diseases usually called spasmodic, unless in cases, where it has lasted so long as to involve other trains of morbid actions, connected with organic lesion; or with a febrile affection of the sanguiferous system. *As its fatal termination has been stated to be effected by a suspension of the natural, and a gradual cessation of the vital functions, so its favorable termination may be stated to be simply a restoration of these functions; a change, which usually takes place with considerable rapidity, and which often arises under circumstances apparently the most desperate.*

[*Diagnosis.*] The diagnosis in cholera is seldom involved in any considerable difficulty, or obscurity. The most important distinction is that between the two species of cholera, the cholera biliosa, and the cholera asphyxia, especially that form of the latter, which is primarily attended with symptoms of excitement. Where the evacuations are tinged of a yellow, or greenish hue, where the matter vomited is bitter to the taste, while the skin remains warm, and the pulse good, the disease may be regarded as bilious cholera, commonly so called:—but, where, after the first emptying of the primæ viæ, the evacuations are of a watery consistence, colourless, turbid, or white; when no urine is voided; where the surface becomes cold; where the features are collapsed; where the spirits are greatly depressed; and where the pulse quickly flags, the case may almost certainly be regarded as cholera asphyxia. As the disease advances, the cessation of the pulse in the arteries of the extremities, the shrivelled and corrugated skin of the hands and feet, the restlessness, deafness, and general depression, leave no doubt of the nature of the disease. Many affections denominated nervous, such as syncope, cholic, hysteria, dyspepsia, spasm of any kind, and the cold stage of fevers are apt, during the prevalence of cholera, to create an alarm of it. The remedies applicable to such cases being in general equally appropriate to the treatment of incipient cases of cholera, and their effects in both instances being often very similar, it is much to be suspected that many of the former have been improperly pronounced to be cases of the latter. Cases of cholera sometimes, apparently, commence by an insidious diarrhœa; or supervene on the action of purgatives, especially saline purgatives; and are then exceedingly apt to be mistaken, both by the patient and his physician. All the experience which we have yet had leaves the mind much in doubt whether this diarrhœa be a primary symptom, or merely indicates a predisposition to the disease. The same observation applies to the effect of purgatives. In such difficult cases, much may be inferred from the state of the epidemic influence prevailing at the time. If cholera be prevalent, they will generally attract immediate notice, and it is the safest course to treat them as cholera: but many of our most lamented casualties have happened from seizures of this description, which were solitary, and altogether unsuspected by the sufferers till too late. There seems however to be something peculiar to cholera, in blinding the patient to the real nature of his case: or, perhaps, conscious of the tendency of some of his symptoms, he seeks to repress the conviction; and is unable to admit or believe that, with little sensible disturbance of health, he already stands on the verge of his grave.

[*Appearances on Dissection.*] The appearances on dissection, after death following attacks of cholera have been very extensively ascertained, in the bodies of European subjects, but in an extremely limited degree in those of native subjects. This is generally stated to have arisen from the aversion betrayed by the friends of deceased natives to the operation of dissection, as if this aversion were peculiar to them. The truth however rather seems to be, that when a European soldier dies, it is extremely rare, that there is any person so connected with him as to derive the right of objection to a post-mortem examination: while every native has friends at hand to see the last offices performed to his body. It is probable that were the cases similar in this respect, we should hear as much of the objections of Europeans, as of natives to dissection of the dead bodies of their friends and relations.

Although dissections are very generally practiced in European hospitals in this country, they are performed under some disadvantages, which must operate to a certain extent, in diminishing the minuteness and accuracy of the information thence derived. The heat of the climate imposes the necessity of interment at a very early period after death; and it likewise imposes the necessity of interring at certain hours of the day, either soon after sunrise, or about sunset. Hence, if a man dies at any time between noon and sunset, his corpe is generally interred the following morning; if he dies any time between sunset and noon, the body is generally buried in the following evening. It follows therefore that there is often but a very limited time allowed for dissection; the pressure of these circumstances however not unfrequently leads to the operation being performed so immediately after death as to afford considerable advantages. Dissections have been chiefly made on the bodies of European soldiers, a class of men acknowledged to be peculiarly liable, in this climate, to visceral disease of all kinds. Under these circumstances, dissection reports should be viewed with care, in reference to the general states of morbid bodies; and with the most attentive consideration of the precise import of the terms employed.

The external appearance of European subjects, who have sunk under cholera, closely resembles that which has been noticed as taking place during life. The surface is livid, the solids are shrunk, the skin of the hands and feet is corrugated. There seems no sufficient evidence of any uncommon tendency in the body to putrefaction after death, nor of any characteristic fœtor from the abdominal cavity. No particular morbid appearances have been found in any of the cavities of the body, which are lined with *serous membranes*, or in these membranes themselves. The cavities of the pleura, of the pericardium, and of the peritoneum have, almost uniformly, been found in a natural state; or, the deviations from that state have manifestly had no connexion with cholera. The surfaces which are lined, or covered with *mucous membranes*, have, on the contrary, very generally exhibited signs of disease. These will be noticed, as the organs connected with them come to be mentioned.

The lungs have not unfrequently been found in a natural state, even in cases where much oppression of respiration had existed previously to death. Much more generally, however, they have been found either to be gorged with dark blood, so that they have lost their characteristic appearance, and have assumed more that of liver, or spleen; or they have been found to be in the opposite state; that is, collapsed into an extremely small bulk, and lying in the hollow on each side of the spine, leaving the cavity of the thorax nearly empty. This appearance has been so remarkable as to induce Dr. Pollock, of H. M. 53d regiment, to conceive, that it could only be produced by the extrication of a gas within the cavity of the pleura, capable of overcoming the atmospheric pressure. It is understood, however, that opportunities were had of piercing the thorax of the dead body under water, and that no gas was extricated. As there appears to have been an absolute vacancy in the cavity of the pleura, that is to say, the lungs did not by any means fill it, it would seem that that viscus had exerted a contractile power, adequate to overcome the pressure of the atmosphere. The blood found in the lungs has been always very black. The heart and its larger vessels have been found to be distended with blood, but not so generally as the apparent feebleness of their propelling power, and the evident retreat of the blood to the centre, would have led us to expect. The right auricle and ventricle being gorged with blood is nothing peculiar to cholera; but some dissections have shewn the left cavities to be filled even with *dark* or *black* blood, which we may reckon as a morbid appearance more peculiar to it. In the abdominal cavity, the peritoneal coverings of the viscera, being *serous membranes*, present in general but little deviation from the healthy state; occasionally, indeed, the morbid accumulation of blood in the vessels of the viscera, imparting an appearance of turgidity and blueness, is evident on their exterior surfaces. We also find them bearing marks of inflammation, especially where the patient may have lingered long, before death. In other cases, the whole tube has had a blanched appearance, both externally and internally. The stomach and intestines generally preserve their ordinary volume. The appearance of the omentum is not sensibly affected in cholera. The stomach is found to be so variously affected as to destroy all grounds for pathological reasoning. It is very rarely found empty, or much contracted after death, nor has any appearance of spastic stricture of the pylorus been often detected. It has however sometimes occurred. Its contents appear to be chiefly the ingesta in an unaltered state: in some cases greenish, or yellow, or turbid matters are found. The stomach has been said to have been found "lined with calomel." Various appearances, either of active inflammation or a congested state of the vessels, have been noticed, sometimes in one part and sometimes in another. The parts seem as if they were sphacelated, thickened, softened, and friable; and, in short, exhibit so great a variety of appearances, from a perfectly natural state to the most morbid, that no particular light is thrown by them on the disease.

The intestinal tube is sometimes collapsed, but oftener found to be more or less filled with air; distended in some parts into bags or pouches, containing whitish, turbid, dark, or green-coloured fluid, and in others, presenting the appearance of spastic constriction. The latter, however, is not common. No fœcal or other solid matters are found in the intestines; but, very commonly, large quantities of the conjee-looking fluid, or of turbid serous matter. The duodenum, and occasionally the jejunum, have been found loaded with an adherent, whitish, or greenish mucus; at other times they have been found seemingly denuded of their natural mucus: and often perfectly healthy. Traces of bile in the intestines, or of any substance apparently descended from the stomach are exceedingly rare. Sanguineous congestion, and even active inflammation, are stated to be more common in the bowels than in the stomach; but on the other hand, instances are very numerous, where no such indications have been detected. The thoracic duct is stated to have been empty of chyle. The liver has been commonly found to be gorged with blood, but not always: it is usually an organ very vascular: and it would probably demand a nicer discrimination than has been bestowed on the subject, to distinguish the degree of congestion in which it is naturally left by the settling of the blood after death in ordinary diseases, from that which has been observed after an attack of cholera. The gall-bladder has almost universally been found to contain bile, and in the great majority of cases even to be completely filled with it. As is usual with this secretion in cases of retention, it is of a dark colour. Very different states of the gall-ducts have been described; cases of constriction and impermeability, seeming to be equally numerous with those of an opposite character.

The urinary bladder is found, we may say universally, without urine, and very much contracted. The lining or mucus membranes of the bladder and ureters, have been found coated with a whitish mucus fluid. The smallness of the bladder after death has been generally adduced in proof of great spasm; but it is not unfrequently found to be equally small after death from other diseases; and, it seems the nature of that organ, when it contains no urine to contract, so as to leave no cavity. Dr. Baillie, in his Morbid Anatomy, thus notices this fact. "The bladder is also found contracted to such a degree as hardly to have any cavity. This is generally not to be considered as a disease, but simply as having arisen from a very strong action of the muscular coat of the bladder previously to death." The appearance of the spleen, which is so various under the ordinary conditions of the body after death, has indicated nothing that can be mentioned as belonging to cholera. The vessels of the mesentery have been very generally found to be uncommonly full of blood.

In the head, appearances of congestion, and even of extravasation have been frequently observed; but, not so uniformly, nor to such extent as to require any particular notice. Only one case has been given, where the state of the spinal marrow was examined: and in that, indications of great inflammation were detected in its sheath: the case, however, was, in some degree, a mixed one,

From this general view of the appearances found on dissection of the bodies of persons, who have died from cholera, it is manifest, that the information thence derivable, is, in a pathological view, of a negative nature only. It is nevertheless, of consequence in a practical sense, especially in treating the sequelæ of cholera.

*(To be concluded in next Number.)*

TABULAR ANALYSIS of the INDIVIDUAL REPORTS transmitted to the MADRAS PRESIDENCY.

| *Name of Medical Officer.* | *Premonitory Diarrhœa.* | *General Symptoms.* | *Consecutive Fever.* | *Contagious or not?* |
|---|---|---|---|---|
| 1. Ass. S. Haines | None mentioned | vomiting and purging, prostration of strength, spasms, languid circulation, sunken eyes, &c. no blueness mentioned | none mentioned | not contagious |
| 2. M'Andrew... | none ditto | the usual symptoms (no blueness) | none | decidedly not contagious |
| 3. Rogers...... | none ditto | the usual symptoms, no blueness mentioned | none | no mention of contagion |
| 4. W. Smith.... | none; restlessness, vertigo, nausea, borborygmi | the usual symptoms, no blueness | none there was sometimes diarrhœa as a sequela | epidemic & not contagious—one attack does not secure against another |
| 5. Sutton...... | none | the usual symptoms, no blueness | none | decidedly non-contagious |
| 6. Scott........ | none | usual symptoms no blueness | none | decidedly non-contagious |
| 7. Sevestrie .... | none | usual symptoms no blueness | none | epidemic |
| 8. White........ | none | usual symptoms no blueness | none | suspects it to be contagious |
| 9. Wyllie....... | none | usual symptoms instantaneous—no blueness | none sometimes disordered secretions of the digestive organs | not contagious |
| 10. W. Ogilby.... | none | usual symptoms no blue colour | none | contagion mentioned |
| 11. Kelly........ | commenced with diarrhœa then vomiting & all the other symptoms in rapid succession | usual symptoms no blue colour | none | endemic and contagious |
| 12. Peyton ...... | none | usual symptoms no blue colour | none | poison in the atmosphere |

| *Name of Medical Officer.* | *Premonitory Diarrhœa.* | *General Symptoms.* | *Consecutive Fever.* | *Contagious or not?* |
|---|---|---|---|---|
| 13. Campbell .... | none | usual symptoms no blue colour, except blue eyelids | none | non-contagious |
| 14. Shedden. .... | none | usual symptoms no blue colour | none | no mention of contagion—considers the disease "more of a diarrhœa than cholera" |
| 15. Dean........ | none; constipation | usual symptoms livid lips | none | epidemic no opinion as to contagion |
| 16. Christy...... | none | usual symptoms no blue colour | none | no mention of contagion |
| 17. Wilson...... | none | usual symptoms no blue colour | none | cannot imagine the cause |
| 18. Boyd........ | no diarrhœa, vertigo, &c. | usual symptoms nails purple | none | no mention of contagion |
| 19. Neilson...... | none | usual symptoms no blue colour | none | no mention of contagion |
| 20. Scarman. .... | none | usual symptoms no blue colour | none | no mention of contagion |
| 21. Millar....... | none | usual symptoms purple colour | none | no mention of contagion |
| 22. Cox. ........ | none | usual symptoms no blue colour | none | no mention of contagion |
| 23. Stone ....... | no diarrhœa, head-ache and languor | usual symptoms no blue colour | none | morbific influence of the atmosphere |
| 24. Colclough.... | none | usual symptoms no blue colour | none | no mention of contagion |
| 25. Trotter...... | none | usual symptoms no blue colour | none | no mention of contagion |
| 26. Duncan...... | none | usual symptoms countenance and nails livid | none | doubtful "causa latet" |
| 27. Orton ....... | giddiness, head-ache, nausea, and griping, loose stools, first symptoms | usual symptoms no blue colour mentioned | no fever mentioned | convinced it is not contagious |

| *Names of Medical Officers.* | *Premonitory Diarrhœa.* | *General Symptoms.* | *Consecutive Fever.* | *Contagious or not?* |
|---|---|---|---|---|
| 28. Owen ....... | none | usual symptoms no blue colour | none | no mention of contagion |
| 29. Mac Laine ... | none | usual symptoms no blue colour | none | no mention of contagion |
| 30. Connell ..... | none | usual symptoms no blue colour | none | unable to give decided opinion, inclines to contingent contagion |
| 31. Bucke ...... | none | usual symptoms no blue colour | none | no mention of contagion |
| 32. Stain........ | none | usual symptoms no blue colour | none | thinks the disease communicable |
| 33. Currie ...... | Diarrhœa in some native soldiers cured without the supervention of cholera | some of the cases had no purging, but constipation. | none | epidemic |
| 34. J. Duncan ... | no diarrhœa | few or no spasms | none | epidemic broke out on the same day that the North-East monsoon set in. |
| 35. Conran...... | no diarrhœa | no cramps or spasm | none | no mention of contagion |
| 36. Goldie ...... | none | usual symptoms | none | decided anti-contagionist. |
| 37. M'Lean...... | none | usual symptoms | none | no mention of contagion |
| 38. Cother ...... | none | usual symptoms no blueness | none | no mention of contagion |
| 39. Neilson...... (Aug. 1821.) | none | usual symptoms no blueness | none | no mention of contagion |
| 40. Mitchell .... (1819.) | none | usual symptoms no blueness | none | not contagious |
| 41. Chalmers.... (1819.) (1820.) | none | usual symptoms the cattle were affected with a similar disease no blueness | none | not contagious travellers appear to carry with them an infectious atmosphere |

| *Names of Medical Officers.* | *Premonitory Diarrhœa.* | *General Symptoms.* | *Consecutive Fever.* | *Contagious or not?* |
|---|---|---|---|---|
| 42. England .... (1819.) | Nausea, diarrhœa, headache, for some hours, and sometimes a whole day | usual symptoms no blueness | none | not contagious |
| 43. Wyse........ (1819.) | none | usual symptoms; fear a great cause no blue colour | none | epidemic |
| 44. Mather ..... (1819.) | none | usual symptoms no blue colour | none | no mention of contagion |
| 45. Wight ...... (1822.) | none | usual symptoms no blueness | dysentery in several cases | epidemic thinks it may be slightly contagious |
| 46. R. Wight .... | none | usual symptoms livid face, and livor of dead body | none | a malignant epidemic |
| 47. Turnbull .... (1822.) | none | usual symptoms no blueness | none | no mention of contagion |
| 48. Donaldson .. (1822.) | none | usual symptoms | none | attributes the disease to various causes, moral and physical. Does not mention contagion |
| 49. R. H. England (1821.) | diarrhœa of some hours, pyrexia | usual symptoms livid appearance | inflammation of brain stomach & bowels sometimes succeeds | assimilates cholera with yellow fever, plague, and typhus, and consequently considers it so far contagious |
| 50. Chapman ... (1821.) | none | usual symptoms no blueness | none | poison in the atmosphere, contagious, afterwards says it is endemic |
| 51. Provan...... (1819.) | none | usual symptoms no blueness | none | inclined to contagion |
| 52. Ewart ...... (1820.) | none | usual symptoms few spasms no blueness | none | cannot divine the cause |
| 53. Williams .... (1820.) | none | skin warmer than usual | none | no opinion |
| 54. Fasken...... | none | usual symptoms | some cases of dysent. and hepat. | non-contagious |

| *Names of Medical Officers.* | *Premonitory Diarrhœa.* | *General Symptoms.* | *Consecutive Fever.* | *Contagious or not?* |
|---|---|---|---|---|
| 56. Barton ...... (1821.) | none | usual symptoms | debility and disordered bowels | not contagious |
| 57. Stokes ...... | none; bowel complaints prevailed previously to the breaking out of cholera | usual symptoms three degrees or grades no blueness | visceral affections, "dropsy and some slight fevers" | seemed sometimes to be communicable |
| 58. A. Campbell.. (1820.) | none mentioned | usual symptoms | diarrhœa | atmospheric |
| 59. Pollock...... (1821.) | no diarrhœa | circulation not affected | none | "some extensive cause" |
| 60. Job ......... (1821.) | none | bilious evacuations few died | none | no cause assigned |
| 61. Cruickshank . (1821.) | none | usual symptoms | none | thinks it infectious, but that its laws are extremely dissimilar to those of other *contagions* |
| 62. Alexander.... (1821.) | none | usual symptoms | none | no opinion |
| 63. Patterson.... (1822.) | none | usual symptoms | none | atmospheric and contagious |
| 64. Dr. W. Pollock (1822.) | none | many of the worst cases had neither vomiting, purging, or spasms | none | a peculiar morbid poison unknown |
| 65. Cowan ...... (1823.) | no premonitory symptoms | usual symptoms purple colour. All other diseases disappeared during cholera | none | no opinion given |

SUMMARY.

| | |
|---|---|
| 1 Suspects it to be contagious. | 1 Decidedly contagious. |
| 1 Doubtful. | 2 Infectious. |
| 1 Contingent contagion. | —— |
| 1 Endemic and contagious. | 10 |
| 1 Slightly contagious. | —— |
| 1 Inclined to think it contagious. | |
| 1 Sometimes communicable. | 13 Decidedly not contagious. |

42 make no allusion to contagion. Of these 42, the great majority speak of the disease as an epidemic arising from some *atmospheric or terrestrial* cause; and, as the queries of the Madras Board particularly directed the attention of medical officers to the subject of contagion, we may fairly conclude that, where no allusion to contagion is made, no proofs of it presented themselves to the observers. Mr. Scot, the Secretary of the Madras Medical Board, sums up the opinions of the contagionists and anti-contagionists, and gives no opinion himself on either side.

Without anticipating the decision of the Bengal Board, we may be permitted to extract the following pithy sentence.

"This is the sum-total of the proofs in favour of the contagious nature of the disease in this quarter. *But, in fact, it amounts to nothing*."—BENGAL REP. p. 147.

---

### REMARKS.

The above tabular analysis of the data on which the Madras Board has founded its report, has cost us much labour, and we have executed this task with impartiality and honesty.

The balance in favour of or against contagion, we leave to our readers, and we shall make no remark on that part of the subject, since the determination of this point ought surely to be left to the evidence of our own senses in this country, where the disease is considered to exist. The summary at the end speaks for itself. In this country, and especially in London, the idea of contagion is very generally abandoned.

The great object of our present enquiry, however, is the *"perfect identity"* of the Indian Epidemic with that which now prevails. An impartial examination of the report which we have here put upon record, must convince unprejudiced readers, that the Indian Epidemic bears a very close resemblance to *one stage or form* of the epidemic which has lately prevailed on the Continent, and now obtains in England. An equally impartial examination of this report will bring, we think, a conviction that, if they be the same disease, they are materially different in their general aspect, when the whole train of phenomena is taken into account. In the Indian report, the premonitory diarrhœa is scarcely alluded to, except in two or three instances, and then it is mentioned as taking precedence of vomiting, in the succession of symptoms; and, in only one instance, as lasting some hours, or perhaps a day, before the confirmed asphyxial form presented itself. Let us compare this with the disease as it appeared in the North, and now exists in London. Diarrhœa is not merely the *precursor* of the deadly, the asphyxial cholera, but it is the first stage of the disease itself, and consequently an integral part of it. Dr. Whiting, who has seen much of it in Southwark, declared in a public society, that the epidemic was a *mild one,* in consequence of the disease being diarrhœa in the first stage, and only assuming the asphyxial aspect when the bowel-complaint was neglected. Nay, this very doctrine has been publicly promulgated by the Board of Health, on Tuesday, the 13th March, 1832, in the following words:—"By the word Cholera, is here to be understood a disease characterised by the following symptoms, viz.—a purging and vomiting of fluids, neither feculent nor bilious, with cramps and prostration, to which, *in extreme cases,* are added coldness, shrinking, &c.*

---

* The Editor of this Journal most unhesitatingly pledges himself to the fact, that the above circular conveys a most erroneous idea of the disease now existing in London. He cedes to every member of the profession, as to talent—to no man, as to accuracy of observation. He agrees with the Board that *feculent vomitings* are not to be frequently found in the epidemic disease; but that the premonitory *diarrhœa* is devoid of *bilious* admixtures in the beginning, he most positively denies. Neither the members of the Board of Health, nor the inspectors employed by them, are competent judges on this point. Those only who are in ample private practice, and who see things with their own eyes, can be proper adjudicators. The descriptions given by patients, and especially by pauper patients, are almost wholly erroneous. It is very true that, after a few days, or, in some cases, after a few hours, the evacuations are found to be *watery.* How can they be otherwise, in people who have evacuated all feculent matters.

Thus then, it is clearly established that diarrhœa, as the first stage, or at all events the preliminary state, is the GENERAL RULE, while, in India, it was the exception, and a very rare one—hence, an important difference between the Asiatic and British epidemics, as far as that stage is concerned.

We now come to examine the asphyxial stage of the disease. And here a most important consideration forces itself, at once, upon our attention. In the East, the asphyxial state was the *first* stage—in the West it is the *second*. In Asia, the individual was knocked down, as it were, by the poison—in Europe, he is prostrated, not only by the original poison, but by the exhausting diarrhœa, which precedes and leads to the cold or asphyxial paroxysm. Here then we see the different conditions of the Asiatic and the European, when the asphyxia commences. No wonder that Magendie observed—" Cholera begins where other diseases end." In Asia, the powers of life are stunned or overwhelmed by the poison—here they are exhausted by the *effects* of the poison (or epidemic influence)—the DIARRHŒA. Hence it is that, in India, the struggling victim shewed the most terrific spasms and the most violent vomiting and purging; bearing the loss of twenty, thirty, fifty, sixty ounces of blood, with the greatest benefit—while, in this country, the asphyxial stage supervening on the diarrhœal, the individual presents the features and phenomena of death, resembling, no doubt, the asphyxia of Indian Cholera, but being the collapse of exhaustion, not that of congestion. In India, at the commencement of the asphyxia, the greater part of the fluids, though driven from the surface, were yet in some part of the body. Here the scanty, and perhaps vitiated fluids, which have been furnished by bad food, or spared by inanition, are drained away by the diarrhœa before the asphyxial stage occurs—and there are no good materials to work upon. Under these circumstances, we can be at no loss to account for the great mortality of the disease in Europe as compared with Asia. On the Continent of Europe it was nearly 1 in 2. In England, it has been somewhat less than 1 in 3. In India, it was not 1 in 7. But this calculation applies to the mortality in the *asphyxial stage* of the disease, both in India and Europe. If we apply the calculus to the

---

and who are unable to take, or unable to procure any ingesta that would produce feculent materials?—" Ex nihilo nihil fit." Those who are accustomed to examine the dejections of people who are labouring under diarrhœa or dysentery, know well that the dejected matters, *after a certain period*, are devoid of either bile or fæculence; they are, in *this*, as in *all* other cases of the kind, and at all other times, *watery*. In forty-nine cases of the epidemic diarrhœa, which the Editor has carefully observed in the middling and upper classes of life, there was fæculence, more or less, and also bilious colouring in the stools. He challenges the whole profession, in this metropolis, to shew him a single case where the "*rice water*" evacuations are the *primary symptoms*, as stated by the Board of Health—and, as to the *ulterior* evacuations, they are watery, have been always watery, and cannot be otherwise than watery, where food cannot be taken, or cannot be procured. The Editor would ask the advocates of " rice water," whether the food (if taken) is converted into gruel;—and if no food is taken, whether there can be any thing but " rice water?"—The plain and evident fact is, that, after the epidemic choleric fever commences in the form of diarrhœa, the patients either have no appetite for food, or have no food to take, in the generality of cases; and as the disease is, to use the words of Sydenham—" a fever turned in upon the guts," all the fluids rush towards that point, and "rice water" is the result. In the middling and upper classes, where the disease is mild, and the appetite and digestion not entirely withdrawn, we have both fæculence and bile in the evacuations. Want of accurate observation is the bane of the medical profession; and there are now, as in the days of Cullen, more false facts than theories in medical matters.—ED.

whole disease, as it appears in this country, and consequently including the diarrhœa, as the Board of Health has officially, and we think properly done, the mortality is not 1 in 50—perhaps not 1 in 100. In respect to the asphyxial stage in this country, under the circumstances above-mentioned, the wonder is, not that the number of recoveries should be so few, but that there should be any recoveries at all!

And here we would draw the attention of our brethren to the danger of applying the Indian practice indiscriminately to the asphyxial stage (the only one where the symptoms resemble each other) of the present epidemic. They are directing the same energetic measures to the *second* stage of a disease, which were employed in India in the *first* stage of a disease said to be identical! Let them ponder on this point! The fatal doctrine of contagion, importation, Asiatic cholera, &c. which has superseded the wise maxims of Sydenham, in studying accurately the constitution of the year, and the phenomena of the reigning epidemic, has led them into a false position. We unhesitatingly maintain, from observation of the disease, both in India and in this country, that, granting, for argument's sake, the Cholera, so called, to be identical in both places—nay more, that it has been transmitted, like a chest of indigo, from Bengal to London;—it is yet, in its asphyxial form, presented to us here, under a totally different character, physiological and pathological. In India, the cold or asphyxial stage was *primary*, and the effect of an intense poison, whatever that poison may have been.—In England, the cold or asphyxial stage is *secondary*, the conjoined effect of an epidemic influence, and of a preceding stage—DIARRHŒA. In the *former* case, the body is *prostrated*, but not drained of its fluids;—in the *latter*, the body is *collapsed*, and the fluids already reduced exceedingly by a flux of considerable duration from the bowels. It is futile to say that there are *exceptions* to this statement. It is by the general rule that we are to be guided, and not by the exceptions. *Exceptio probat regulam.*

### CONSECUTIVE FEVER.

We now come to a most important point of the inquiry, as to the identity of the Asiatic and English epidemics—namely, the consecutive fever. In respect to the MADRAS Medical Board, we need only refer to the report itself to prove that the word *fever* is not even mentioned. The recovery is compared to that from *syncope*, where proper means are employed; and, under other circumstances, the sequelæ are reported as chronic visceral affections. No such thing as *fever* is alluded to. But referring from the aggregate account of the Board to the individual reports, from which the account was drawn, we find that not one single reporter enumerates *fever* as a sequela of cholera, in the extensive territory under the Presidency of Madras! To this we may add the testimony of Sir William Russell himself, who officially stated the fact, that in the disease, as it appeared in Russia, an entirely new feature was added—the consecutive fever—which destroyed more than the original disease. Is it possible that human reason, or even inveterate prejudice can require more than this evidence to bring conviction that FEVER was no consecutive or integral part of Indian cholera, as far as the Presidency of Madras is concerned?

We now turn to Bombay. The Medical Board of that Presidency quotes a passage from Mr. Jukes, to the following effect, as relates to BENGAL.

"The fever which invariably *attended* this second stage of the disease, may be considered to have been rather the result of Nature's effort to *recover herself from the rude shock* which she had sustained, than as performing *any integral and necessary part* of the disorder itself. It partook much of the common *bilious attacks* prevalent in these latitudes." xix.

The evident and natural conclusion is, that what Mr. Jukes calls the *fever*, is the reaction from the cold stage, like the hot, or fever stage of an ague. But be this as it may, the following comment by the Bombay Board will settle the question, as far as the Bombay Presidency is concerned.

"We shall only therefore mention, that the subsequent fever, which it appears has generally *accompanied it in Bengal,* has been but little, *if at all,* observed on this side of India." xxi.

We think we have now demonstrated that throughout the whole peninsula of India, namely, the Presidencies of Madras and Bombay, the consecutive fever, so universally observed on the Continent and in England, was absent. If these differences do not destroy the "perfect identity" of the disease, as it presented itself in Asia and Europe, then there is an end to all fair reasoning in medical science.

But we will add one more argument against the "*perfect identity*" of the disease in India and Europe, which will place some of our opponents between the caudine forks. It will hardly be disputed that the practitioners of India were better judges of the *appearances of cholera* there, than our literary practitioners at home, who never saw the disease. What says the Bombay Board then, as to the identity of the disease *in India* and the disease in England, as described by Sydenham ?

"This disease seems to have been known to Dr. Sydenham, and to have been *accurately described by him as a prevailing epidemic in England in* 1669, *under the title of Cholera Morbus* ; and as he no where mentions bile as forming any part of the discharges from the stomach or bowels, it may be justly inferred that if it had, it could not have escaped the notice of so accurate an observer. He says—

'Malum ipsum facile cognoscitur, adsunt enim vomitus enormes, ac pravorum humorum cum maxima difficultate et angustia alvi dejectio; cardialgia, sitis. Pulsus celer ac frequens, cum æstu et anxietate, non raro etiam parvus et inæqualis, insuper et nausea molestissima, sudor interdum diaphoreticus, crurum et brachiorum contractura, animi deliquium, partium extremarum frigiditas, cum aliis notæ symptomatibus, quæ astantes magnopere perterrefaciant, atque etiam angusto viginti quatuor horarum spatio ægrum interimant.'

And again in his letter to Dr. Brady describing the epidemics of 1674, 5, and 6, he says—

'Exeunte æstate Cholera Morbus epidemice jam sæviebat, et insueto tempestatis calore evectus, atrociora convulsionum symptomata, eaque diuturniora secum trahebat, quam mihi prius unquam videre contigerat. Neque enim solum abdomen, uti alias in hoc malo, sed universi jam corporis musculi, brachiorum crurumque præ reliquis, spasmis tentabantur dirissimis, ita ut æger e lecto subinde exiliret, si forte extenso quaquaversum corpore eorum vim posset eludere.'

The first of these extracts describes the disease with great accuracy as it, very generally, affected the Natives—the second is well exemplified in Dr Burrell's report as it attacked the Europeans of the 65th Regt. at Seroor." *Rep.* xxiii.

Now the advocates of "perfect identity" deny that the disease in this country at present, at all corresponds with that of Sydenham ; while the Bombay Board asserts that Sydenham's description exactly applies to the cholera of India. See, then, in what a dilemma the party in question is placed. The Bombay Board proves the identity of the *Indian* cholera and Sydenham's. The party alluded to, denies that the malady here is at all identical with, or similar to, the disease of 1669 ! By every law of logic then, and by their own shewing, the Indian and the English epidemics are not "perfectly identical."

To place this dilemma in as concise and clear a point of view as possible. The epidemic described by Sydenham is identical with the epidemic of India, according to the Bombay Board.—The epidemic of India is identical with that now existing in England—ergo, Sydenham's epidemic of 1669, and the reigning epidemic of 1832, are identical. *Q. E. D.* We defy the advocates of "perfect identity" to extricate themselves from this dilemma, except by denying the competency of the Bombay Board to appreciate the nature of the disease which was around them.

We think we have abundantly proved that wherever the disease originated, or however it may be produced, the character of it in this country is very different from that which it assumed in India. There it was cholera rather than fever—here it is fever rather than cholera. It is not a little curious that, after all the cry that has been raised about the cholera *travelling* from India to England, the compliment is now returned by the Bombay Board, who assert that the cholera which scourged India in their day, was the English cholera of 1669!! So that, long as was the time which the malady took to come from the Ganges to the Thames, it must have taken a still longer period to travel from England to Bengal.

These are some of the consequences of adopting the notion that epidemics travel from one country to another, instead of the more philosophical doctrine that the elements of epidemic diseases exist in all countries, and are called into activity, simultaneously or successively, by a cause, of which we have no knowledge, and over which we have no control.

But when we deny the "*perfect identity*" of the Indian and English epidemics, we do not mean to deny that the same epidemic *causes* have originated somewhat similar diseases in India, Continental Europe, and England. We only maintain that neither the causes nor the diseases themselves *travelled* from one country to another; but *occurred* as much spontaneously in Bombay as in Bengal—in Persia as in Hindostan—in Russia as in Persia—in Prussia as in Russia—in Hamburgh as in Riga—in Sunderland as in Hamburgh—in Glasgow as in Newcastle—in London as in Musselburgh.

In respect to the doctrine of the disease being a *nova pestis*, in any of the countries above-mentioned, the evidence put on record by the Madras Board pretty clearly settles that question in the negative. But we will introduce a document from the pen of Frank, which must silence the most strenuous advocate for a new disease. The document is extremely valuable on many accounts, and bears with intense interest and importance on the present epidemic.

### EXTRACTS FROM FRANK ON THE CHOLERA.*

| Text. | Translation. |
|---|---|
| I. "Sporadice hic morbus sub cœlo temperato, ac nequidem frequenter occurrit; rarius, interdum epidemice; calidioribus in locis frequentius grassatur et quasi endemicus agnoscitur." 249. | This disease occurs sporadically and not very frequently in temperate climates; sometimes, but more seldom, as an epidemic; in warmer countries, it rages and assumes an endemic character. |

### II. On the Connexion between Cholera and Fever.

| | |
|---|---|
| "Majoris in medicæ artis exercitio momenti divisio est choleræ in apyrecticam quæ sine febre incedit; et in febrilem, quæ febris legitimæ, periodicæ, et quidem perniciosæ, plerumque tertianæ, symptoma est (febris intermittens cholerica), ac loca *paludosa*, calido sub cœlo, vel maxime infestat; cum prima, ubique frequentius hac ipsa occurrat." 250. | "A division of cholera, of greater importance in the practice of medicine, is that into the apyrectic, or the cholera unaccompanied with fever; and the febrile, which is a symptom of a genuine, periodical, most commonly tertian, and truly pernicious fever (intermitting choleric fever), which chiefly infests marshy situations in hot climates; the first variety is the more common of the two." |

### III. Description of an Attack of Cholera.

| | |
|---|---|
| "Subito et cum impetu plerumque cholera hominem prehendit. In qui- | The cholera commonly attacks the patient with suddenness and violence. Sometimes |

* De Curandis Hominum Morbis, &c. Lib. 6tus, p. 249 *et seq.* Editio Viennæ Austriæ, 1807.

busdam eandem præcedunt sensus lassitudinis, stomachi tensio, dolores, ructus acidi nidorosi, nausea, salivæ frequens exspuitio, abdominis inflatio cum strepitu, colici dolores pungentes. Mox uno impetu et tempore, vel saltem alternatim et vomitus et fluxus alvi effrenes sequentur. Primum aquæ similia, dein, acsi recens caro lota esset—non nunquam alba et nigra,—in aliis, quod rarum est, lympha modo limpida, et ad frigus coitura, —in multis, primum ciborum reliquiæ, postea humores biliosi, plus nimus muco permisti, mox flavi, mox æruginosi, mox fusci, nigri, plerumque acidissimi ac fere corrosivi una cum copiosis iterum ructibus et flatibus, necnon ipso non nunquam cum sanguine, repetitis frequentissimè vicibus, et sub tam subitanea virium prostratione redduntur, ut assumti veneni haud raro subintret suspicio. Ventriculus interea ipsaque intestina mirum contorqueri solent in modum. *Vehementissimi illum scilicet cardialgia, cum siti intensa, voce clangosa et rauca,*—hæc vero, *maxime suprà umbilicum rosionis, morsicationis sensus invadit. Urina in vesica multorum retinetur,* aut certe urit excreta. Ad anum, tenesmus urget. Singultus fortiori malo succedit; *crurum, surarum, brachiorumque musculi tenduntur,* convelluntur; *incurvantur digiti, livent ungues, algent extrema, frigido viscidoque sudore obtecta, cum internorum ardore,* ac animi deliquiis. *Arteriæ jam ante hac contractæ ac minimæ, pulsationes nunc offerunt frequentissimas, inordinatas; nunc vero a digito vix amplius distinguendas.* Vehementiore in cholera, ægroti, ob immensam adeo, ac celerem humorum ex ventriculo intestinisque simul jacturam, ob crudelice et animi et corporis tormenta, *post quinque, sexve jam horas, faciem vel maxime mutatam, collapsam, et sibi dissimilem ostendunt,* atque nisi promptum feratur auxilium, nunc adeo angusto unius nycthemeri spatio,—nunc secunda, tertia quartâve die, raro tardius; efflant animam." 250-1.

we find these premonitory symptoms: a sense of lassitude, tension, and pain of the stomach, acid and fetid eructations, nausea, frequent spitting, flatulence and borborygmi, pungent colicky pains. After a time, violent vomiting and purging set in, sometimes together, sometimes alternating with each other. At first the egesta are like water; then as if flesh had recently been immersed in them, —sometimes they are white, sometimes dark, —in others, which is uncommon, they are merely a limpid lymph—in many cases, the discharges are, first, the remains of the food that has been taken, afterwards fluids of a bilious character, more or less mixed with mucus, now yellow, now æruginous, now dusky, dark, often very acid and almost corrosive, with copious eructations, sometimes even blood, very frequently repeated, and with such sudden prostration of strength, that not uncommonly a suspicion of poison having been taken presents itself. The stomach, in the interim, and the intestines are convulsed in an extraordinary manner. The patient is affected with the most severe pain at the scrobiculus cordis, intense thirst, a shrill raucous voice, and with a great sensation of gnawing or erosion at the umbilicus; tenesmus is urgent. The urine in many cases cases is retained, or if passed is hot and irritating. As the disease advances, hiccup succeeds—the muscles of the thighs, legs, and arms are seized with spasms—the fingers are flexed and contracted, the nails are livid, the extremities are cold, and covered with a cold, clammy sweat, whilst the internal parts feel burning, and there is syncope. The pulse, previously contracted and very small, now becomes frequent and irregular, now no longer to be felt. In the worst cases of cholera, the patients, exhausted by the profuse and rapid discharge of humours from the stomach and intestines, and by their cruel torments, mental and corporeal, exhibit in five or six hours a remarkable change in their features, which are *collapsed, and no longer like themselves.* Unless the patient is speedily relieved, he sinks in the space of twenty-four hours, or on the second, third, seldom later than the fourth day.*

* The following passage, from Torti, lib. iii. chap. i. will not be uninteresting at the present moment. Speaking of a certain form of pernicious fever, of a remittent type, he observes:

"Circa paroxysmi *initium*, vehemens contingit, violentaque, ac simultanea excretio sursum ac deorsum humorum vitiosorum, prava insimul qualitate, atque immodica quantitate peccantium, sive *sinceri* illi sint, sive variegati: quibus nonnunquam vomitibus, ac dejectionibus copiosis, et crebris, singultus adjungitur, *vox rauca, oculorum concavitas, angor stomachi, pulsus exilis, extremosum perfrigeratio, aut livor:*—uno verbo, *omnia accidentia quæ choleram morbum comitari solent, &c.*"

If the above be not a choleric fever, and very analogous to the present epidemic, we are incapable of any discrimination.

The foregoing extracts will supply the deficiency in the terse account of Sydenham and other writers. Frank's account embraces almost every feature and phenomenon of the epidemic cholera, or rather choleric fever, as described by Asiatic, Continental, or English writers.* But we particularly invite the attention of our professional brethren to Frank's remarks, as to the connexion of cholera with fevers of the intermitting and remitting character, in low and malarious situations. Many practitioners of this metropolis are now perceiving, that the choleric fever of the present period is frequently ushered in with rigors or cold chills, and observes a periodical character, where the first collapse or cold stage does not prove fatal, which is too often the case. Several of them have found that the vomiting and purging recur at certain periods, and that bark effectually stops the paroxysms. This is a point of most vital importance, and we urgently request our brethren to attend to it carefully.

* Great stress is laid on the nature of the ejected matters in cholera. If the fluids are colourless, it is a proof that the disease is Asiatic—if any bile or other coloured fluids appear, the disease is English. Yet the India Boards, and, indeed, all careful observers, have acknowledged that the discharged fluids form no criterion of the disease. The Bengal Board, for example, tells us that "the fluid ejected from the stomach was watery; mostly tasteless, transparent, or of a whey or ash colour. Sometimes it was *sour, green, dark,* like infusion of tea, starchy, mixed with mucus, and viscid. In very rare cases, pure bile was thrown up." In respect to the alvine evacuations, they were "generally watery, colourless, white, or muddy;—*sometimes red and bloody, sometimes greenish and pulpy,* like half-digested vegetables." Is it not preposterous, after this, to make the distinction between Asiatic and English disease to consist in the colour of the motions? Nimium ne crede colori!*

* Mr. Ekin, a learned young surgeon, has pointed out in Areteus some passages, which have been erroneously construed by a writer in the Medical and Physical Journal. Thus, in speaking of the treatment of cholera, Areteus says: "Hæc vero facienda sunt *donec* stercora per inferiora dejicientur, et superius biliosa efferantur." "These means are to be pursued UNTIL (not "*so long as,*" as has been represented) fæcal and bilious matters appear." This, we think, settles the point as to Areteus. This ancient author states the suppression of urine in cholera, and takes pains to account for this symptom.

